# Role of USP7 in the regulation of tolerogenic dendritic cell function in type 1 diabetes

**DOI:** 10.1186/s11658-025-00727-5

**Published:** 2025-04-17

**Authors:** Farhan Ullah Khan, Puregmaa Khongorzul, Denis Gris, Abdelaziz Amrani

**Affiliations:** 1 Department of Pediatrics, Immunology Division, Université de Sherbrooke Faculté de Médecine et des Sciences de la Santé, 3001, 12 th Avenue North, Sherbrooke, QC J1H 5 N4 Canada; 2 Department of Phamacology-Physiology, Université de Sherbrooke Faculté de Médecine et des Sciences de la Santé, 3001, 12 th Avenue North, Sherbrooke, QC J1H 5 N4 Canada

**Keywords:** Type 1 diabetes, Dendritic cells, USP7, Ezh2, PD-L1/2, Immunotherapy

## Abstract

**Background:**

Tolerogenic dendritic cells (toDCs) are critical for maintaining immune homeostasis and preventing autoimmune disease development, such as type 1 diabetes (T1D). We have previously shown that DCs of non-obese diabetic (NOD) mice expressing active Stat5b (Stat5b-CA.DCs) acquire toDCs signature and protect against diabetes. However, the mechanisms involved in reprogramming DCs to adopt tolerogenic or immunogenic signatures are not fully known. This study investigates for the first time the role of USP7 in DC-mediated immune regulation in T1D using a transgenic NOD mouse model expressing an active form of Stat5b (NOD.Stat5b-CA).

**Methods:**

Splenic DCs were purified from diabetes-prone NOD mice and diabetes-resistant NOD.Stat5b-CA transgenic mice and their tolerogenic and immunogenic phenotypes were analyzed by FACS. Their pro-and anti-inflammatory cytokine patterns, IRF4, IRF8, de-ubiquitin ligase USP7, and methyltransferase Ezh2 expression were assessed by FACS and Western blot. Moreover, the impact of USP7 inhibition in DCs on Th1/Th2/Th17 and Treg and diabetes onset was assessed using an in vivo DC-based transfer model.

**Results:**

In this study, we found that splenic Stat5b-CA.DCs expressed high levels of USP7, Ezh2, and PD-L-1/2 and contained a higher proportion of tolerogenic conventional DC2 (cDC2) subsets than immunogenic cDC1 compared to NOD mice DCs. We also found that the USP7 blockade increased Stat5b-CA.DCs maturation and proinflammatory cytokines production while decreasing anti-inflammatory cytokines and PD-L1 and PD-L2 expressions. Mechanistically, USP7 blockade in Stat5-CA.DCs promoted cDC1 over cDC2 subsets by increasing IRF8 expression in an Ezh2-dependent manner and decreasing IRF4 expression independently of Ezh2. USP7 blockade also increased Stat5b-CA.DC capacity to promote Th17 and to restrain Th2 and Treg cells. Importantly, the capacity of Stat5b-CA.DCs to protect NOD mice from diabetes were lost when treated with USP7 inhibitor.

**Conclusions:**

Our findings underscore the role of the USP7/Ezh2 axis in maintaining tolerogenic DC functions that are required to tailor adaptive immune response and diabetes protection in NOD mice.

**Graphical abstract:**

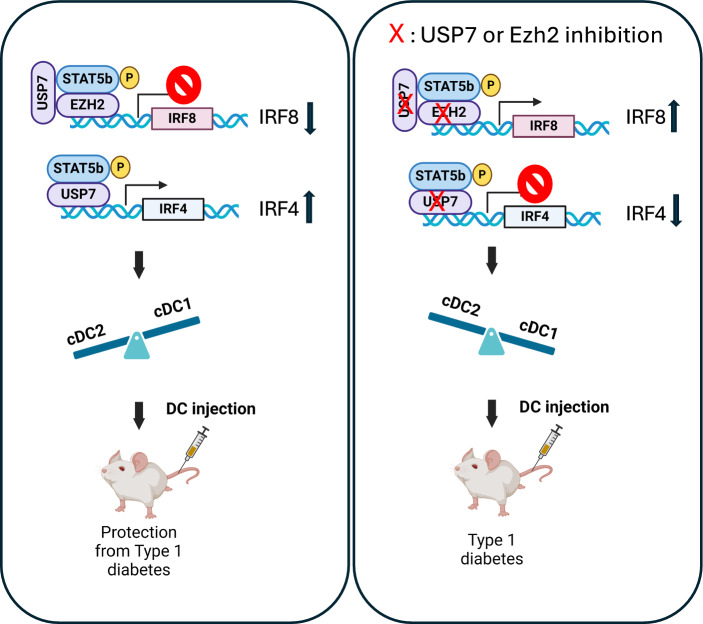

**Supplementary Information:**

The online version contains supplementary material available at 10.1186/s11658-025-00727-5.

## Introduction

Type 1 diabetes (T1D), a chronic autoimmune disease, results from lymphocyte-mediated destruction of islet-specific β-cells leading to insulin shortage and hyperglycemia [1]. Studies in T1D patients and non-obese diabetic (NOD) mice, an animal model that mirrors many features of human T1D, have shown that CD4^+^ and CD8^+^ T cells play an important role in β-cell destruction, and that islet-specific autoreactive CD8^+^ T cells are the ultimate effector in β-cell attack [2]. Besides T lymphocytes, antigen-presenting cells (APCs) such as dendritic cells (DCs) have also been found among immune cells infiltrating the islets and are described as major immunoregulators of the adaptive autoimmune response in T1D and many other autoimmune diseases [3–5]. Since, under normal conditions, targeted pancreatic β-cells do not express MHC II but express low levels of MHC I molecules, the pathogenic autoreactive T cell activation and effector function are more likely to result from contact with β-cell antigens on APCs such as DCs.

DCs are professional APCs that play an important role in directing T-cell immune responses and are among the first APCs homing to islets and constitute the major APCs of islet infiltrates that trigger primary T-cell responses to β-cell antigens [6]. T-cell activation status is facilitated by DC phenotypic and functional changes in response to inflammatory signals, like pathogen-associated molecular patterns (PAMPs) and damage-associated molecular patterns (DAMPs). Under the inflammatory condition, DCs upregulate surface co-stimulatory markers (CD80 and CD86), express high levels of MHC molecules, augment chemokine receptors (e.g., CCR7) expression to facilitate their migration to the lymph node, and secrete inflammatory cytokines such as IL-12 [7]. Depending on the type of the inflammatory or anti-inflammatory signal and the transcriptional factors involved in the signaling pathway, DCs acquire the properties of immunogenic or tolerogenic DCs. Tolerogenic DCs are shown to serve as mediators of immune tolerance to prevent the development of autoimmunity [8]. They utilize several mechanisms to establish and sustain peripheral immune tolerance, including T cell anergy induction, promoting regulatory T cells (Tregs) differentiation, and reducing or depleting effector T cells. Although the precise mechanism leading to the breakdown of islet antigen tolerance is not fully understood, the fact that DCs are among the first immune cells homing to the NOD pancreatic islets suggests that they play a crucial role in the initiation of the islet inflammatory autoimmune response [9]. Several studies have revealed numerous DC phenotypic and functional abnormalities in T1D in NOD mice, such as expressing high levels of costimulatory molecules and producing large amounts of proinflammatory cytokines [10, 11]. In addition, DC depletion completely prevented the onset of islet inflammation (insulitis) and diabetes in the NOD mice [12]. To overcome the abnormalities of DCs that are the major contributors to the development of autoimmune responses leading to diabetes in NOD mice, we engineered a transgenic mouse model called NOD.Stat5b-CA mice in which DCs express an active form of Stat5b transcription factor (Stat5b-CA.DCs) [13]. We found that Stat5b-CA.DCs exhibited the characteristics of tolerogenic DCs and these mice were fully protected against diabetes. Furthermore, we observed that Stat5b-CA.DCs led to the induction of autoimmune tolerance by enhancing the number and suppressive activity of Tregs, as well as promoting CD4^+^ T cell differentiation towards Th2 and Tc2 immune responses [13]. These tolerogenic properties of splenic DCs were also acquired when DCs were derived from bone marrow precursors of NOD.Stat5b-CA mice [14].

DC progenitors arise from bone marrow precursors, circulate in the blood for a few days, and then migrate into the tissues, where they mature into various DC subsets. In lymphoid organs and non-lymphoid tissues of mice, conventional DCs (cDCs) can be largely classified as cDC1 and cDC2 subsets. cDC1, considered as immunogenic DCs, can be distinguished by the expression of CD8α^+^/XCR1^+^/CD11b^−^ and their generation is dependent on the expression of transcription factors IRF8 and BATF3 [15]. cDC1 function resides in their capacity to execute strong cross-presentation of viral antigens, a phenomenon related to chemokine receptor XCR1 expression that results in effective priming of CD8^+^ T cells [16]. They can also recognize intracellular pathogens and induce type 1 immune responses, such as the induction of the Th1 response, ILC1, and NK cell activation [17]. On the other hand, the cDC2 subset, also known as tolerogenic DCs, represents the major subpopulation of DCs in blood and is identified by the expression of CD4^+^/CD11b^+^/SIRPα^+^ [15]. Their differentiation involves mainly a transcriptional factor IRF4, but other TFs are also required [14]. cDC2 s have been shown to induce Th1, Th2, and Th17 responses [18, 19]. They are also effective inducers of Tregs [20]. However, how immunogenic (cDC1) and tolerogenic (cDC2) DC differentiation and function are regulated is far from being fully understood.

Ubiquitin-specific proteases (USPs) are the largest subfamilies of deubiquitinases (DUBs), of which USP7, also named herpes-associated ubiquitin-specific protease (HAUSP), is one of the most prominent and well-characterized DUB members [21]. Since its discovery, several lines of evidence have shown that the deubiquitylating enzyme USP7 regulates various physiological processes and interacts with numerous cellular pathways. USP7 has attracted considerable interest as an anticancer target as demonstrated by its major role in tumorigenesis, cancer metastasis, and promoting cancer cell proliferation by stabilizing the Ki-67 protein [22, 23]. USP7 is also implicated in other cancer-related targets, including PTEN, N-Myc, p53, PHF8, TRIP12, and ASXL1 [24, 25], thereby regulating different critical biological processes such as epigenetic regulation [26], DNA damage repair [27], neuronal dendritic growth [28] and immune responses [29]. Notably, USP7 stabilizes the expression of a master regulator of Tregs FOXP3 as well as other Tregs transcriptional factors such as Tip60, p300, and DNMT1 as well as TGF-β, which are essential for Tregs differentiation, development, and stabilization [26, 30, 31]. Tregs play a central role in the regulation or suppression of immune responses to infectious pathogens and malignancies, as well as to self-antigens, allergens, and commensal microbiota [32]. More recently, it was shown that USP7 is involved in regulating plasmacytoid DCs (pDCs) phenotype and function in multiple myeloma (MM) [33]. However, the contribution and the role of USP7 in tolerogenic DCs and in type 1 conventional DCs (cDC1) and type 2 conventional DCs (cDC2) phenotype and function have not yet been investigated.

In this study, we report the first experimental evidence that tolerogenic splenic Stat5b-CA.DCs express high levels of ubiquitin protease USP7, Ezh2, and PD-L-1/2 and contain a higher proportion of cDC2 than the cDC1 subset compared to splenic DCs of NOD mice. We also found that the inhibition of USP7 resulted in tolerogenic Stat5b-CA.DCs activation/maturation, less expression of PD-L1/2, and reprogramming them to produce more proinflammatory but less anti-inflammatory cytokines. Notably, USP7 and Ezh2 inhibition restrained the tolerogenic cDC2 population by downregulating IRF4 expression in an Ezh2-independent manner while promoting the immunogenic cDC1 population by upregulating IRF8 expression in an Ezh2-dependent manner. We further demonstrated that the adoptive transfer of USP7 inhibitor-treated DCs into prediabetic NOD mice reduced the populations of Tregs and Th2 cells and their IL-4 and IL-10 anti-inflammatory cytokine production while fostering the function of Th17 cells by upregulating the production of IL-17 cytokine. Moreover, blockage of USP7 in tolerogenic Stat5b-CA.DCs abrogated their capacity to protect NOD mice from developing diabetes.

## Materials and methods

### Mice

The transgenic NOD.Stat5b-CA mice have been generated in our laboratory as we described previously [13]. NOD mice were from the Jackson Laboratory (Bar Harbor, ME, USA). All animal care and experimental procedures complied with the standards outlined in protocol number 2022–3652 and adhered to the University of Sherbrooke's institutional animal care guidelines, as well as the Canadian Council on Animal Care (CCAC) standards. The mice were housed under specific pathogen-free conditions with unrestricted access to food and water. Gender and age-matched animals were used.

### Dendritic cell preparation and treatment

Splenic DCs were purified using a commercial DC purification kit (cat#130-125-835, Miltenyi Biotec, San Diego, CA, USA). Briefly, spleens were isolated from NOD and NOD.Stat5b-CA mice, digested with collagenase-D (1.5 mg/mL) at 37 °C for 40 min, and single-cell suspension was prepared following the manufacturer’s instructions. The splenic cells were then incubated with CD11c Microbeads and maintained at 4 °C in the dark for a duration of 10 min. Following incubation, DCs were washed with phosphate-buffered saline (PBS) containing 0.5% bovine serum albumin (BSA) and EDTA (2 mM). Subsequently, the samples were processed through a magnetic separation column to isolate magnetically labeled cells. Finally, the yield of CD11c^+^ cells was assessed by flow cytometry for DC purity. In all experiments, the purity of the cell was ˃ 90% (Supplementary Fig. 1).

After isolation, purified DCs were cultured in LCM media composed of RPMI 1640 medium, 10% low endotoxin fetal bovine serum (FBS), penicillin (100 U/mL), streptomycin (100 µg/mL), and β-mercaptoethanol (50 µM). DCs were then treated with or without USP7 inhibitor (P5091) (5 µM/mL) for 24 h. This dose and duration of treatment were selected based on previous studies [34, 35] and our preliminary MTT assay. In some experiments, splenic DCs were incubated with LCM media containing either vehicle (0.1% DMSO) or Ezh2 inhibitor GSK343 (3 μM/mL) alone or together with the USP7 inhibitor P5091 (5 μM/mL) for 24 h. Thereafter, cells were harvested, washed with PBS, and used for the assays as described in figure legends.

### Antibodies and FACS analysis

Cell FACS analysis was conducted as described in our previous work [13]. In brief, DCs were labeled with the following fluorochrome-conjugated anti-mouse antibodies, purchased from either eBiosciences, Life Technologies, or BioLegend: anti-CD11c-APC (clone N418; cat# 117310), anti-CD11b-Pacific Blue (clone M1/70; cat# 101224), anti-MHCII-PE (clone 10-3.6; cat# 109908), anti-CD40-PE-CY5 (clone 1 C10; cat# 15–0401-82), anti-CD80-PE (clone 1G10; cat# A14723), anti-CD86-PE-Cy7 (clone GL1; cat# A15412), anti-PD-L1-PE (clone MIH5; cat# 12–5982-82), anti-PD-L2-PE (clone TY25; cat# 12–5986-82), anti-XCR1-Brilliant Violet 785 (clone ZET; cat# 148225), anti-Sirp-α-PerCp-efluor710 (clone P84; cat# 46–1721-8), anti-CD4-APC-efluor 780 (clone RM4-5; cat# 47-0042-82), and anti-CD8-Percp-CY5.5 (clone 53-6.7; cat# 45–0081-82).

For the assessment of intracellular IRF4, IRF8, Ezh2, and USP7 protein expression, DCs were first incubated with anti-cell surface marker antibodies for 30 min in the dark. Thereafter, cells were collected, washed with PBS, fixed with 4% paraformaldehyde (PFA) for 40 min, and then permeabilized using the Foxp3 Staining Kit (eBioscience, San Diego, California, USA) for 35 min. After permeabilization, cells were incubated with anti-IRF4-PE (clone 3E4; cat# 12–9858-82), anti-IRF8-APC (clone V3GYWCH; cat# 2093671), or with anti-Ezh2 (cat# 4905S, Cell Signaling Technology), and anti-USP7 primary Ab (cat# 4833, Cell Signaling Technology, Whitby, Ontario, Canada). Thereafter, cells were stained with a secondary Alexa Fluor 488-anti-rabbit IgG antibody (cat# A-21206, Invitrogen, San Diego, California, USA). Finally, DCs were washed and acquired with CytoFLEX instrument (Beckman Coulter, Brea, CA, USA). The resulting data were processed and evaluated with FlowJo software version 10.2 (Tree Star Inc., Ashland, OR, USA).

### Western blot

Protein extraction was performed by lysing DCs in the lysis buffer (cat# 9803S, Cell Signaling Technology, Boston, MA, USA) containing protease and phosphatase inhibitors. Proteins were then fractionated on SDS-PAGE gels (10%) and transferred onto PVDF membranes (Millipore, QC, Canada). Membranes were then blocked with 5% dry milk and incubated overnight at 4 ℃ with an anti-USP7 primary antibody (cat# 4833, Cell Signaling Technology), followed by an appropriate secondary antibody (cat# 7074S, Cell Signaling Technology). β-actin (cat# 8457, Cell Signaling Technology) was used as a loading control. To visualize protein bands, enhanced chemiluminescence (GE Health Care Canada Inc., Oakville, ON, Canada) was used.

### Cytokine production and quantification

For cytokine quantification, DCs pretreated with or without USP7 inhibitor were cultured in the presence or absence of lipopolysaccharides (LPS) (1 µg/mL). After 24 h, the quantification of cytokines (TNF-α, IL-1β, IL-6, and IL-10) released in the supernatants was assessed using the Meso Scale Discovery (MSD) assay (MSD U-PLEX Platform, Rockville, Maryland, USA). TGF-β was quantified using an ELISA kit (ThermoFisher Scientific, Waltham, MA, USA) following the protocol provided by the manufacturer.

### Dendritic cell injection and T-cell response monitoring

Purified splenic DCs isolated from NOD and NOD.Stat5b-CA mice were preincubated with or without USP7 inhibitor (5 µM) for 1 h and then washed with PBS prior to stimulation with LPS (1 µg/mL). After 24 h, DCs were harvested, washed, and intravenously injected (6 × 10^6^ cells/mouse) into prediabetic NOD mice [8–10 weeks old]. After 7 days, recipient mice were euthanized, and spleen T cell subsets were analyzed by flow cytometry. Briefly, splenic cells were incubated with anti-CD4-APC (clone GK1.5; cat# 17–0041-82), fixed with 4% PFA and permeabilized, and then intracellularly stained with anti-Foxp3-Fitc (clone FJK-16 s; cat# 11–5773-82), anti-T-bet-PE (clone eBio4B10; cat# 12–5825-80), anti-Gata-3-Percp-efluor 710 (clone TWAJ; cat# 46–9966-42), or anti-RORγt-PE (clone AFKJS-9; cat# 12–6988-82) antibodies. To quantify cytokine production, cells were incubated with Brefeldin A (2 μM; eBiosciences) for 4 h, and stained with anti-CD4 antibodies, before permeabilization and intracellular staining with anti-IL-4-FITC (clone BVD6-24G2; cat# 11–7042-82), anti-IL-10-PE (clone JES5-16E3; cat# 12–7101-82), anti-IFN-γ-APC (clone XMG1.2; cat# 17–7311-82), and anti-IL-17-FITC (clone eBio17B7; cat# 11–7177-81) mAbs. Antibodies used were from eBiosciences (San Diego, CA). Cell acquisition and analysis were performed using flow cytometry as described in the FACS analysis section.

### DC treatment and diabetes monitoring

Splenic DCs isolated from transgenic NOD.Stat5b-CA and NOD mice were not treated or treated with USP7 inhibitor (5 µM for 1 h) before stimulation with LPS (1 µg/mL) for 24 h. Thereafter, DCs were washed and intravenously transfused (6 × 10^6^ cells/mouse) to female NOD mice (3–4 weeks old) and then monitored for diabetes development using glucose readings via Uristix strips (Bayer, Minneapolis, MN, USA). Diabetes onset was confirmed by measuring blood glucose using the Accu-Check Advantage monitoring system (Roche Diagnostics, Indianapolis, IN, USA). Mice were followed for diabetes for up to 32 weeks or until diabetes occurred. Mice tested positive for urine glucose (with Uristix) were considered diabetic when two consecutive blood glucose were higher than 15 mmol/L, mice.

### Statistics

Data were analyzed using GraphPad Prism software version 10.0 (GraphPad Software Inc., La Jolla, CA, USA), and results are presented as the mean ± standard error of the mean (SEM). Where appropriate, the two-tailed unpaired Student’s *t*-test was applied to compare two groups. One-way ANOVA followed by a Tukey multiple comparison test was used for multiple comparisons. For diabetes incidence comparisons, Kaplan–Meier survival analysis with Gehan–Breslow–Wilcoxon test was used. *P* < 0.05 was considered a cutoff for determining statistical significance.

## Results

### Tolerogenic DCs of NOD.Stat5b-CA mice express elevated levels of USP7

**Fig. 1:**
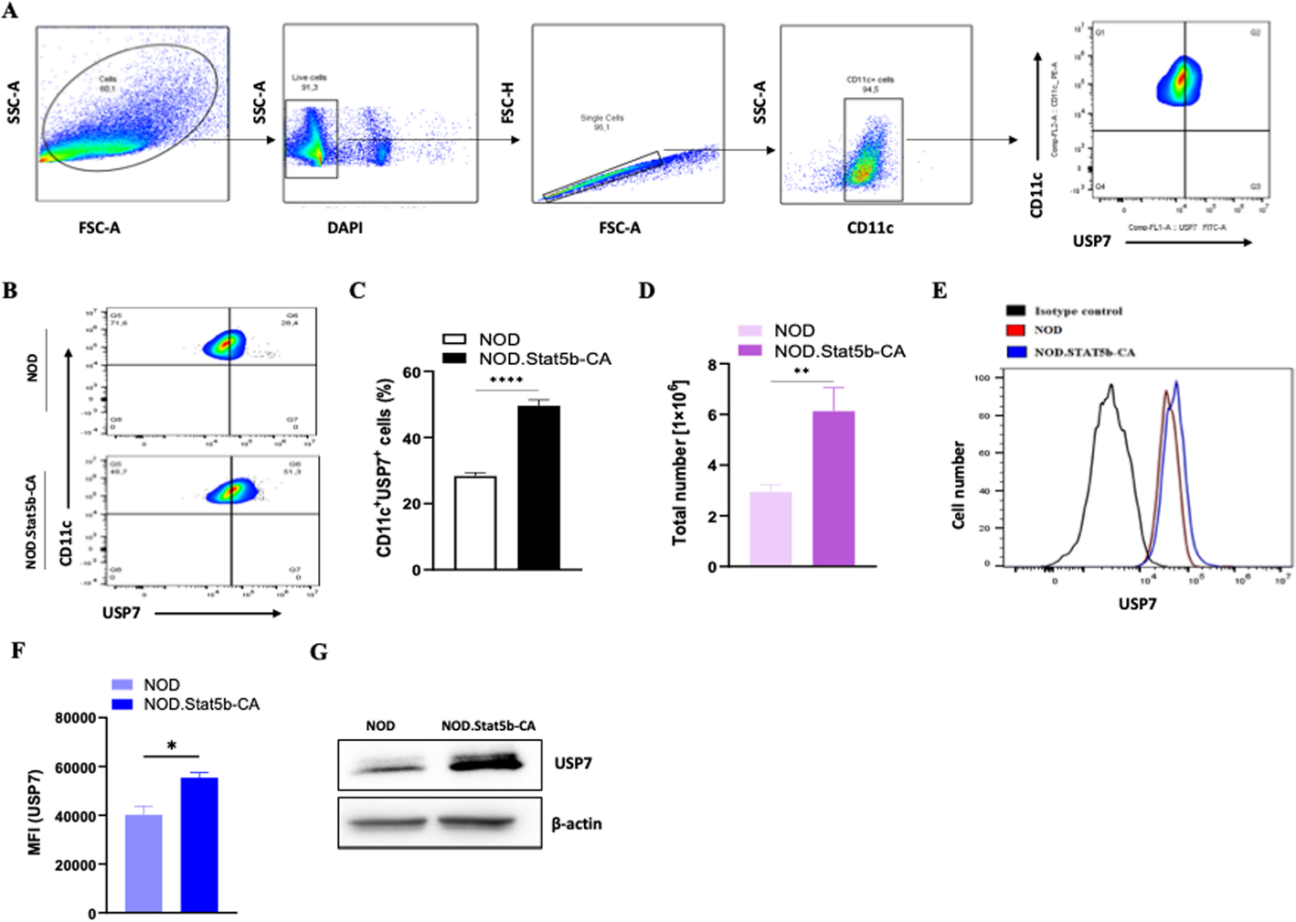
Tolerogenic DCs of transgenic NOD.Stat5b-CA mice express higher USP7 than immunogenic DCs of NOD mice. **A** Representative FACS plots showing gating strategy used for USP7 expression analysis in purified splenic CD11c + DCs from the spleen. **B** Counter plots showing CD11c^+^USP7^+^ cell frequencies in purified splenic DCs of NOD and transgenic NOD.Stat5b-CA mice. **C** Representative bar graph showing USP7 frequency. **D** Absolute numbers of USP7 expressing DCs. **E** Histogram showing USP7 expression level in purified splenic CD11c^+^ DCs. **F** Mean fluorescence intensity (MFI) of USP7 expression and (**G**) Western blot analysis of USP7 expression and β-actin used as a loading control. Data are shown as the mean ± SEM of three independent experiments. The two-tailed unpaired Student’s *t*-test was used. *P < 0.05; **P < 0.01.****P < 0.0001

USP7 expression and function are mostly investigated in various cancers [23, 24] and in Tregs in physiological and pathological conditions, including one study showing a low level of USP7 expression in Tregs of diabetes-prone NOD mice as compared to Tregs from diabetes-resistant mice [30, 31, 36]. However, USP7 expression and function in conventional cDCs have not yet been investigated. We have previously demonstrated that Stat5b.CA expressing DCs of NOD mice exhibit a mature phenotype that are endowed with a tolerogenic function characterized by reduced IL-12 production and increased TGF-β secretion as well as inducting of antigen-specific Th2/Tc2 immune responses and promoting Treg differentiation relative to immunogenic DCs of NOD mice [13]. Therefore, we first explored USP7 expression in immunogenic and tolerogenic splenic cDCs purified from NOD and transgenic NOD.Stat5b-CA mice, respectively. Using the gating strategy for splenic CD11c^+^ DC shown in Fig. 1A, flow-cytometric analysis showed significantly increased frequency and number of CD11c^+^ DCs expressing USP7 in NOD.Stat5b-CA mice as compared to those in NOD mice (Fig. 1B–D). FACS data analysis also showed higher USP7 expression in splenic DCs of NOD.Stat5b-CA mice than in splenic DCs of NOD mice (Fig. 1E, F). High level of USP7 expression in tolerogenic DCs of NOD.Stat5b-CA was further confirmed using western blot analysis (Fig. 1G). These data demonstrate that immunogenic DCs of diabetes-prone NOD mice express a lower level of USP7 than tolerogenic DCs of diabetes-resistant NOD.Stat5b-CA mice.

### Targeting USP7 inhibition promotes tolerogenic Stat5b-CA.DC maturation and disturbs their immunoregulatory cytokine production

**Fig. 2:**
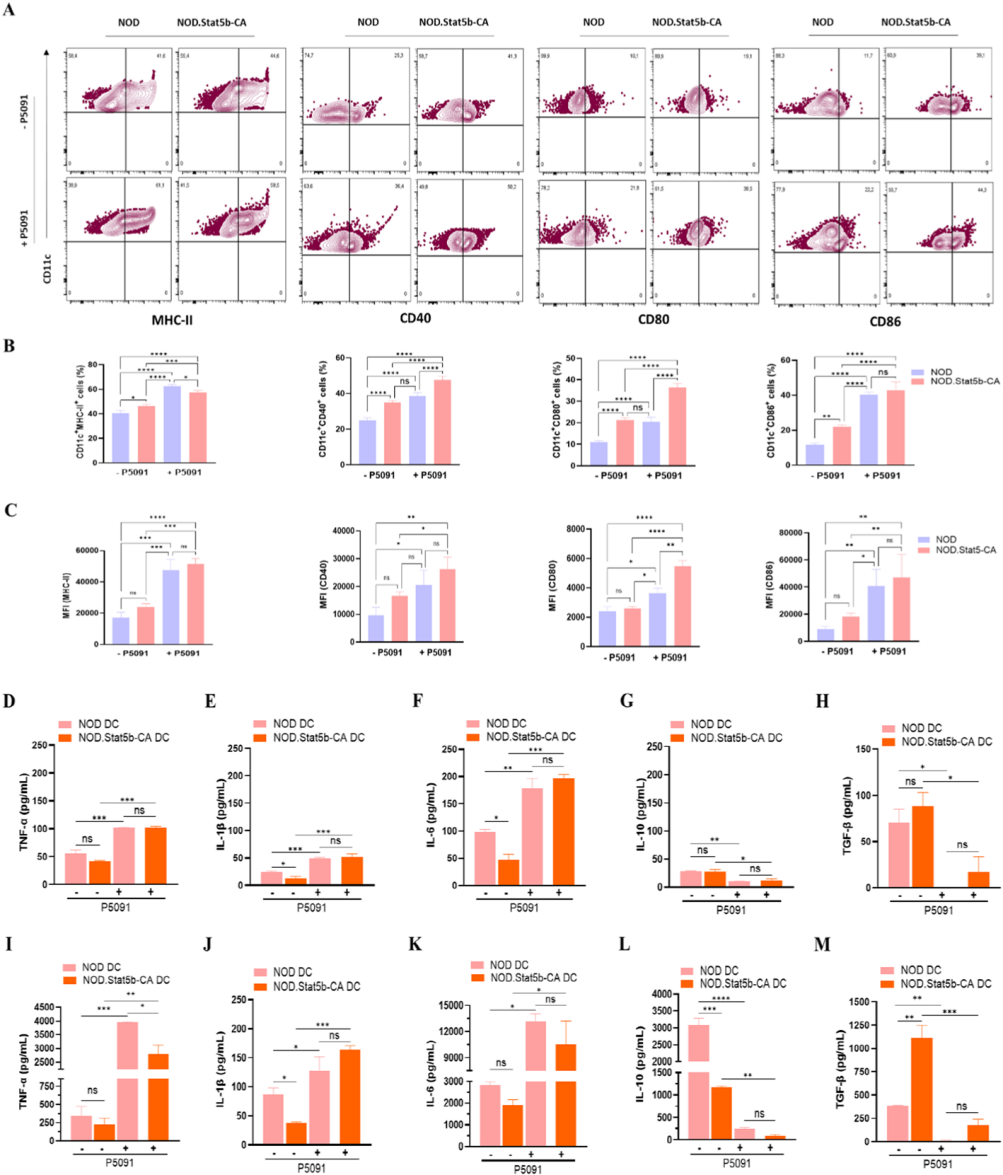
Pharmacological inhibition of USP7 increases DC mature phenotype and facilitates their proinflammatory cytokines production. Purified splenic DCs (1 × 10^5^ cells/well) from NOD and NOD.Stat5b-CA mice were cultured in the presence (+ P5091) or absence (− P5091) of USP7 inhibitor (5 µM/mL) for 24 h. DCs were washed, labeled with anti-CD11c mAbs in combination with anti-MHC class II, anti-CD40, anti-CD80, or anti-CD86 mAbs, and analyzed by FACS. **A** Flow cytometry plots depicted cell surface expression of MHC class II, CD40, CD80, and CD86. **B** Representative bar graphs showing the percentages of positive cells with respect to the total population of purified splenic DCs. **C** Mean fluorescence intensities (MFI) values of FACS profiles. For cytokines quantification, purified splenic DCs (5 × 10^5^ cells/well) from NOD and NOD.Stat5b-CA mice were cultured for 24 h in the presence (+ P5091) or absence (− P5091) of USP7 inhibitor (5 µM/mL) and then stimulated with LPS (1 µg/mL) for an additional 24 h. Quantification of TNF-α, IL-1β, IL-6, IL-10, and TGF-β released in the supernatants of splenic DCs before (**D**–**H**) and after LPS stimulation (**I**–**M**). Data are shown as the mean ± SEM of at least three independent experiments. One-way ANOVA followed by Tukey’s post hoc test was used. n.s, not significant; *P < 0.05; **P < 0.01; ***P < 0.001; ****P < 0.0001

The ability of DCs to induce inflammatory immune responses or promote immune tolerance is directly related to their maturation status and function (cytokine production). To examine whether USP7 inhibition affects the DC maturation, splenic DCs of NOD and NOD.Stat5b-CA mice were not untreated or treated with USP7 inhibitor P5091, which irreversibly targets USP7 activity without affecting cell viability [37, 38]. After 24 h, DCs were analyzed for the expression of activation/maturation and MHC-II markers. FACS data showed increased CD11c^+^ DCs expressing CD40, CD80, and CD86 in NOD.Stat5b-CA mice in comparison to DCs of NOD mice (Fig. 2A, B). Not surprisingly, like our previous report [13], control (vehicle-treated) immature DCs from both NOD and NOD.Stat5b-CA expressed similar levels of MHC-class II, CD40, CD80, and CD86 co-stimulatory molecules (Fig. 2C). Interestingly, USP7 blocked significantly augmented the frequencies of MHC-II, CD40, CD80, and CD86 expressing DCs (Fig. 2A, B) and the expression levels of these markers in DCs of NOD and NOD.Stat5b-CA mice (Fig. 2C). Levels of MHC-II, CD40, and CD86 were similar in the DCs of both strains of mice, whereas the CD80 level was higher in the DCs of NOD.Stat5b-CA mice than in DCs of NOD mice (Fig. 2C). Likewise, the activation of DCs with LPS promoted DC maturation in both strains of mice, as we previously reported [13], and inhibition of USP7 further endorsed their maturation level (data not shown).

Next, we investigated whether the USP7 blockade affects tolerogenic Stat5b-CA.DCs cytokine patterns before and after LPS stimulation. Results showed that bystander production of TNF-α, IL-1β, and IL-6 pro-inflammatory cytokines by unstimulated splenic DCs of transgenic NOD.Stat5b-CA mice were lower than those produced by control NOD splenic DCs (Fig. 2D–F). In contrast, a higher amount of anti-inflammatory cytokine TGF-β was produced by DCs of NOD.Stat5b-CA compared to the NOD splenic DCs, while similar amounts of IL-10 were produced by unstimulated DCs of both strains of mice (Fig. 2G, H). Interestingly, treatment with USP7 inhibitor induced unstimulated splenic DCs to produce significantly higher amounts of TNF-α, IL-1β, and IL-6 but significantly lower amounts of IL-10 and TGF-β as compared to unstimulated DCs pretreated with vehicle (control DCs) (Fig. 2D–H). This cytokine pattern was similar between USP7 inhibitor-treated DCs of NOD.Stat5b-CA and NOD mice. Notably, LPS stimulated Stat5b-CA.DCs produced less amounts of TNF-α, IL-1β, and IL-6, less IL-10 proinflammatory cytokines, but a higher amount of anti-inflammatory TGF-β as compared to LPS-stimulated DCs of NOD mice (Fig. 2I–M). The production of proinflammatory cytokines TNF-α, IL-1β, and IL-6 produced by LPS-stimulated DCs of NOD were further increased, whereas IL-10 and TGF-β production were further decreased when DCs were pretreated with USP7 inhibitor (Fig. 2I–M). Together, these results demonstrate that inhibition of USP7 promotes tolerogenic Stat5b-CA.DCs and immunogenic NOD DCs maturation and reroute tolerogenic NOD.Stat5b-CA.DCs to acquire a marked proinflammatory cytokine profile.

### USP7 inhibition downregulates Stat5b-CA.DC PD-L1 and PD-L2 expressions

**Fig. 3:**
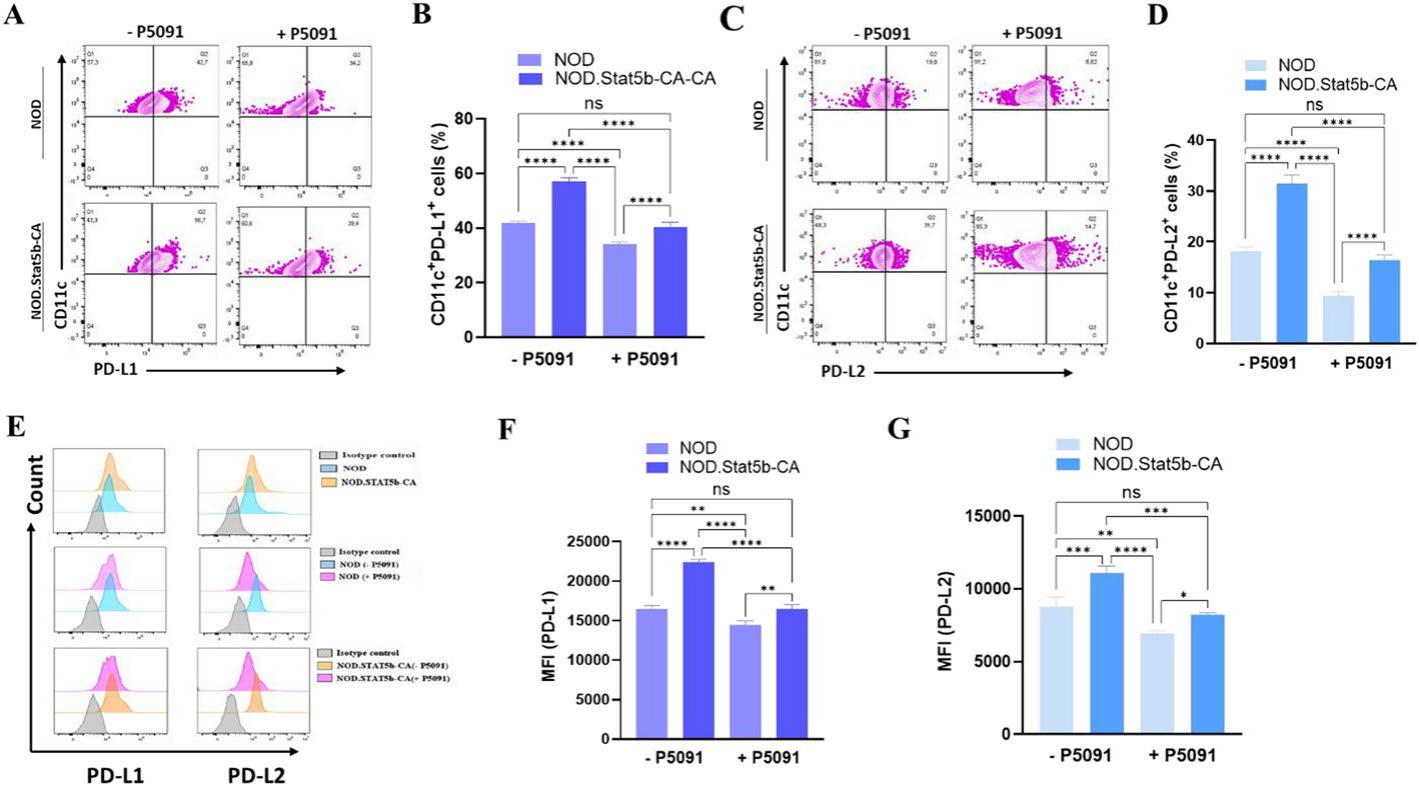
USP7 blockade decreases PD-L1 and PD-L2 expression in tolerogenic Stat5b-CA.DCs. Purified splenic DCs (1 × 10^5^ cells/well) from NOD and NOD.Stat5b-CA mice were cultured for 24 h in the presence (+ P5091) or absence (− P5091) of USP7 inhibitor (5 µM/mL). DCs were washed, labeled with anti-CD11c mAbs in combination with anti-PD-L1 and anti-PD-L2 mAbs. **A**–**D** Flow cytometry plots depict PD-L1 and PD-L2 expression (**A**, **C**) and cell frequencies (**B**, **D**) in CD11c^+^ DCs. **E** Flow cytometry histogram depicts levels of PD-L1 and PD-L2 expressions and (**F**–**G**) MFI in CD11c^+^ DCs treated with or without USP7 inhibitor (5 µM/mL). Data are shown as the mean ± SEM of five independent experiments. One-way ANOVA followed by Tukey’s post hoc test was used. n.s, not significant; *P < 0.05; **P < 0.01; ***P < 0.001; ****P < 0.0001

The above data suggested that inhibition of USP7 in tolerogenic splenic Stat5b-CA.DCs disturb their immunoregulatory phenotype and function (cytokines profile). PD-L-1 and PD-L2 costimulatory molecules are highly expressed by tolerogenic DCs, and their binding to PD-1 results in the inhibition of T-cell proliferation, and inflammatory cytokine production, thereby inducing peripheral immune tolerance. Therefore, we examined whether USP7 inhibition affects the expression of PD-L1 and PD-L2 in tolerogenic Stat5b-CA.DCs and immunogenic DCs of NOD mice. Flow cytometry data showed that CD11c^+^PD-L1^+^ and CD11c^+^PD-L2^+^ DC frequencies were significantly higher in NOD.Stat5b-CA mice compared to those of NOD mice (Fig. 3A–D). Similarly, PD-L1 and PD-L2 expression levels were significantly higher in Stat5b-CA.DCs than DCs of NOD mice (Fig. 3E–G). Importantly, inhibition of USP7 prominently reduced both the frequencies of CD11c^+^PD-L1^+^ and CD11c^+^PD-L2^+^ DCs, as well as PD-L1 and PD-L2 expression levels in DCs of both strains of mice (Fig. 3A–G). Notably, the impact of USP7 blockade on the reduction of PD-L1 and PD-L2 expression was more pronounced in tolerogenic Stat5b-CA.DCs than in immunogenic cDCs of NOD mice (Fig. 3E–G). Together, these data demonstrate that USP7 is also involved in maintaining high levels of PD-L1 and PD-L2 inhibitory receptor expression in tolerogenic DCs of NOD.Stat5b-CA mice.

### USP7 inhibition promotes cDC1 over cDC2 subset in tolerogenic Stat5b-CA.DCs

**Fig. 4:**
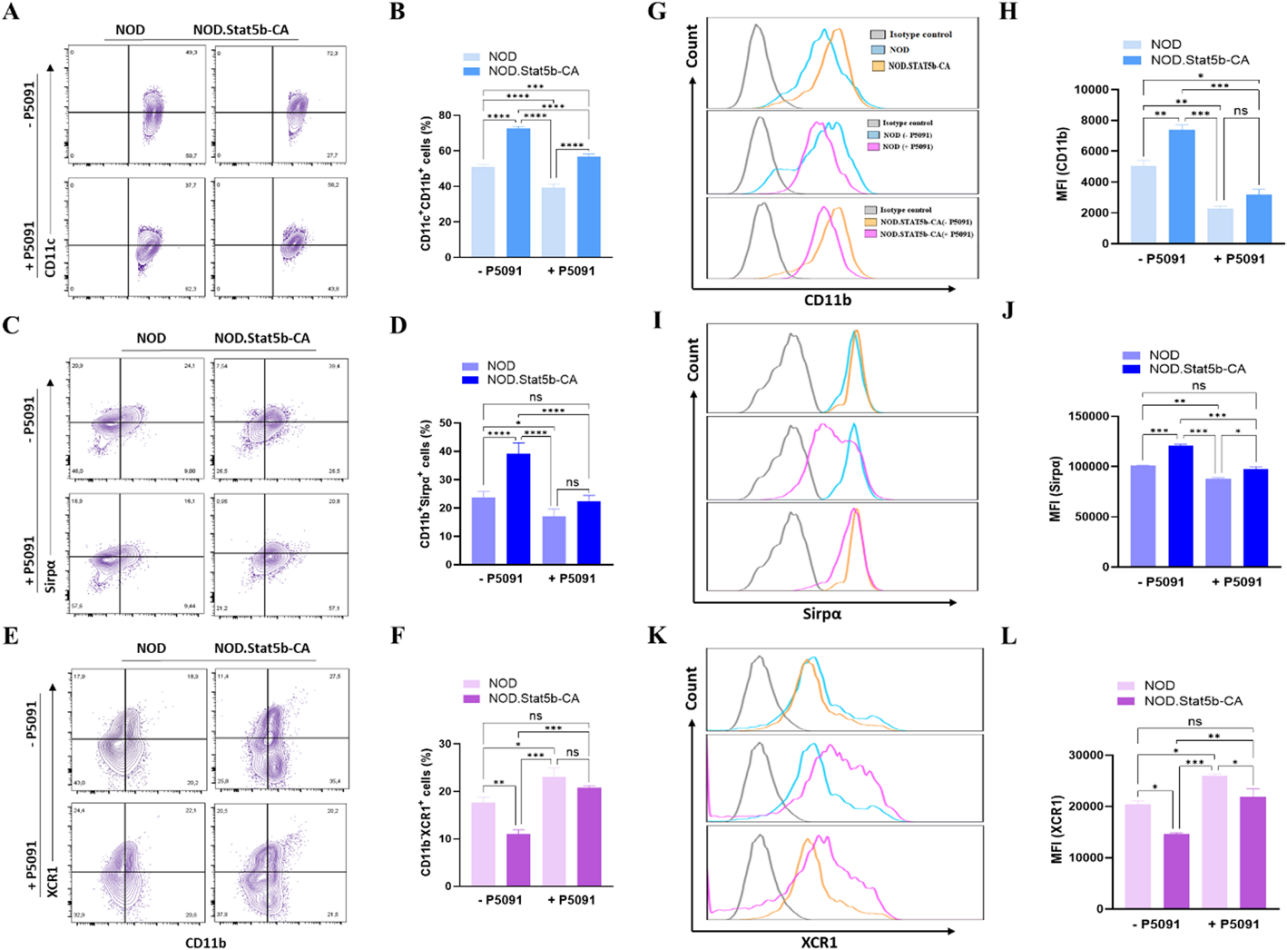
Inhibition of USP7 promotes cDC1 over cDC2 in tolerogenic Stat5b-CA.DCs. Purified splenic DCs (1 × 10^5^ cells/well) from NOD and NOD.Stat5b-CA mice were cultured in the presence (+ P5091) or absence (− P5091) of USP7 inhibitor (5 µM/mL) for 24 h. DCs were washed, labeled with anti-CD11c mAbs in combination with anti-CD11b, anti-Sirpα, and anti-XCR1 mAbs. **A**, **C**, **E** FACS profiles showing CD11b, XCR1, and Sirpa expression in splenic CD11c^+^ DCs treated with or without USP7 inhibitor. **B**, **D**, **F** Representative bar graphs of CD11c^+^CD11b^+^, CD11b^+^Sirpa^+^, CD11b^−^XCR1^+^ cDC frequencies. (**G**,** I**, and **K**) Representative histograms of CD11b, Sirpα, and XCR1 gated on CD11c^+^ as assessed by flow cytometry. **H**, **J**, **L** Mean fluorescence intensity (MFI) values of CD11b, Sirpα (gated on CD11c^+^CD11b^+^ DCs), and XCR1 (gated on CD11c^+^CD11b^−^ DCs) expression. Data are shown as the mean ± SEM of at least four independent experiments. One-way ANOVA followed by Tukey’s post hoc test was used. n.s, not significant; *P < 0.05; **P < 0.01; ***P < 0.001; ****P < 0.0001

The various DC subsets, particularly cDC1 and cDC2, play a crucial role in the induction of immunogenic and tolerogenic immune responses. In mouse autoimmunity, cDC1 was attributed to being immunogenic and cDC2 being tolerogenic. Since NOD.Stat5b-CA mice comprise a higher proportion of splenic CD11c^+^CD11b^+^ cDC2 than the proportion of CD11c^+^CD11b^+^ cDC2 subsets in NOD mice [39], we next investigated whether inhibiting USP7 in splenic DCs would promote immunogenic cDC1 over tolerogenic cDC2 subpopulations in NOD and NOD.Stat5b-CA mice. To this end, purified splenic cDCs were cultured with or without USP7 inhibitor P5091 for 24 h, and cDC1 (CD11c^+^CD11b^−^CD8^+^XCR1^+^) and cDC2 (CD11c^+^CD11b^+^CD4^+^Sirpα^+^) populations were analyzed by FACS. In agreement with our previous data [39], NOD.Stat5b-CA mice displayed an elevated frequency of tolerogenic splenic CD11c^+^CD11b^+^Sirpα^+^ cDC2 subset and a low frequency of immunogenic CD11c^+^CD11b-XCR1^+^ cDC1 cells in comparison to the high frequency of immunogenic cDC1 and low frequency of tolerogenic cDC2 in NOD mice (Fig. 4A–F and Supplementary Fig. 2 A, B). We also found a high level of CD11b and Sirpα expression in cDC2 and a low XCR1 expression level in cDC1 cells of NOD.Stat5b-CA mice. On the contrary, CD11b and Sirpα were less expressed in cDC2 cells, whereas XCR1 was highly expressed in cDC1 cells of NOD mice (Fig. 4G–L).

Intriguingly, USP7 inhibition considerably reduced the frequency of the tolerogenic cDC2 population while significantly augmenting the frequency of the immunogenic cDC1 population in both strains of mice (Fig. 4A–F). Indeed, cDC2/cDC1 ratio in Stat5b-CA.DCs were higher than in NOD DCs and were switched to a low cDC2/cDC1 ratio when treated with USP7 inhibitor (Supplementary Fig. 2 C). The switch in cDC2 and cDC1 ratio induced by USP7 inhibition was also accompanied by a significant reduction of CD11b and Sirpα expression level in cDC2 subsets and enhanced XCR1 expression level in cDC1 subsets (Fig. 4G–L). Altogether, these results reveal that the deubiquitinating enzyme USP7 plays an important role in regulating XCR1^+^ cDC1 and Sirpα^+^ cDC2 subsets in NOD and NOD.Stat5b-CA mice.

### USP7 differentially regulate IRF4 and IRF8 expression

**Fig. 5:**
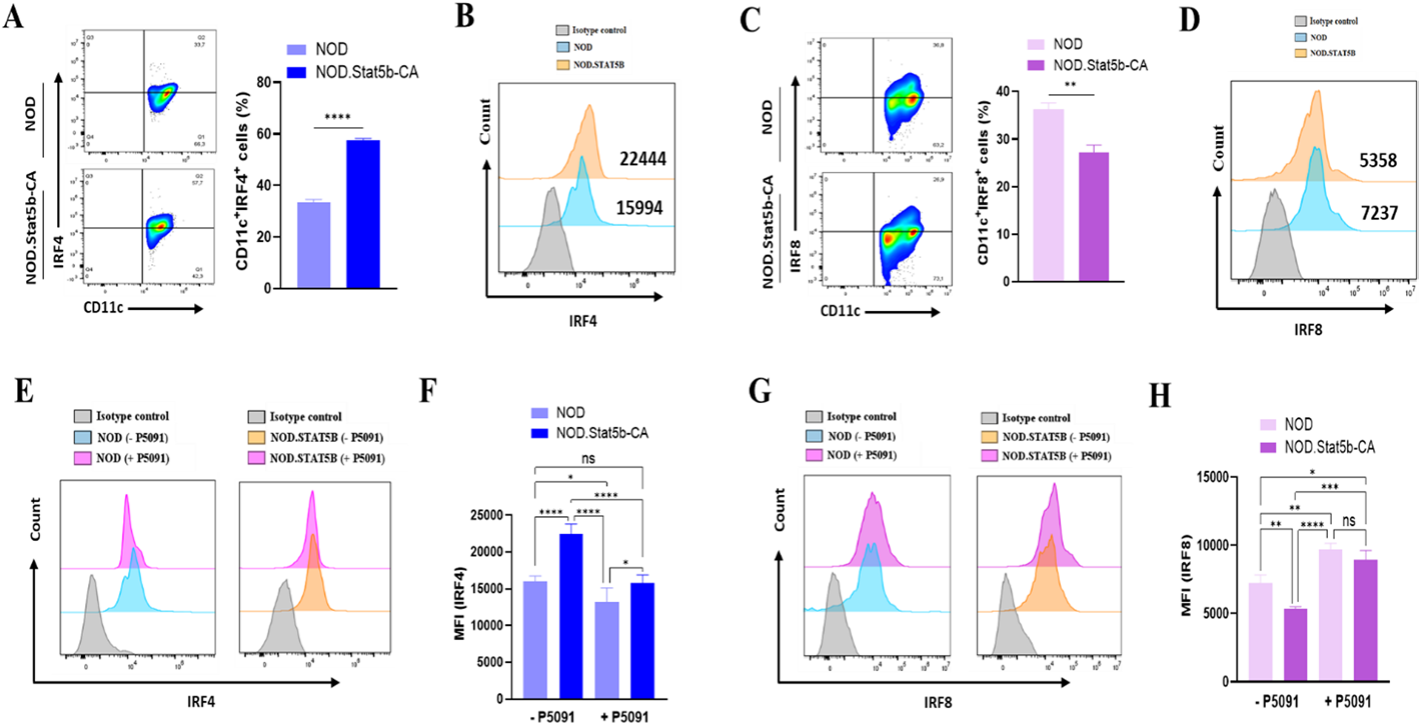
Blockade of USP7 downregulates IRF4 and upregulates IRF8 expression. Purified splenic cDCs (1 × 10^5^ cells/well) from NOD and NOD.Stat5b-CA mice were cultured for 24 h in the presence (+ P5091) or absence (− P5091) of USP7 inhibitor (5 µM/mL). cDCs were washed, labeled with anti-CD11c mAbs in combination with Abs anti-IRF-4 or anti-IRF-8, and analyzed by FACS. **A**, **C** Counter plots (left) and representative bar graphs (right) showing CD11c^+^IRF4^+^ (**A**) and CD11c^+^IRF8^+^ (**C**) cell frequencies in purified splenic cDCs. **B**, **D** Flow cytometry histogram showing IRF4 (**B**) and IRF8 expressions (**D**) in splenic CD11c^+^ DCs. **E**, **G** Flow cytometry histograms showing IRF4 (**E**) and IRF8 (**G**) expression in purified splenic CD11c^+^ DCs treated with or without USP7 inhibitor (5 µM/mL) for 24 h. **F**, **H** Mean fluorescence intensity (MFI) values of IRF4 (**F**) and IRF8 (**H**) expression. Data are shown as the mean ± SEM of five independent experiments. The significance was calculated using One-way ANOVA with Tukey’s post-test. n.s, not significant; *P < 0.05; **P < 0.01; ***P < 0.001; ****P < 0.0001

cDC1 and cDC2 development and function depends on transcription factors IRF8 and IRF4, respectively [40–42]. Recently, we have reported high IRF4 and low IRF8 expression in tolerogenic splenic and bone marrow-derived DCs of NOD.Stat5b-CA mice when compared to NOD mice [14, 39]. Therefore, we explored whether the expression of IRF4 and IRF8 in splenic DCs was regulated by USP7. For this purpose, the levels of IRF4 and IRF8 expression were analyzed in splenic cDCs of NOD and NOD.Stat5b-CA mice before and after USP7 inhibition. FACS results showed a higher frequency of IRF4^+^CD11c^+^ DCs, as well as high expression levels of IRF4 in NOD.Stat5b-CA compared to that of the NOD mice (Fig. 5A, B). On the contrary, the frequency of IRF8^+^CD11c^+^ cDCs and the expression level of IRF8 were higher in NOD mice compared to that of the NOD.Stat5b-CA mice (Fig. 5C, D). Data also showed that USP7 blockade significantly reduced IRF4 expression level while increasing IRF8 expression level in both Stat5b-CA.DCs and NOD DCs when compared to untreated DCs (Fig. 5E–H). Of interest, the reduction of IRF4 expression and the increase of IRF8 expression in the presence of USP7 inhibitor were more prominent in cDCs of NOD.Stat5b-CA mice (Fig. 5E–H). Altogether, these findings highlight the importance of USP7 in regulating IRF4 and IRF8 and, thereby, cDC1 and cDC2 differentiation.

### Ezh2 contributes to IRF8 but not IRF4 regulation through USP7

**Fig. 6:**
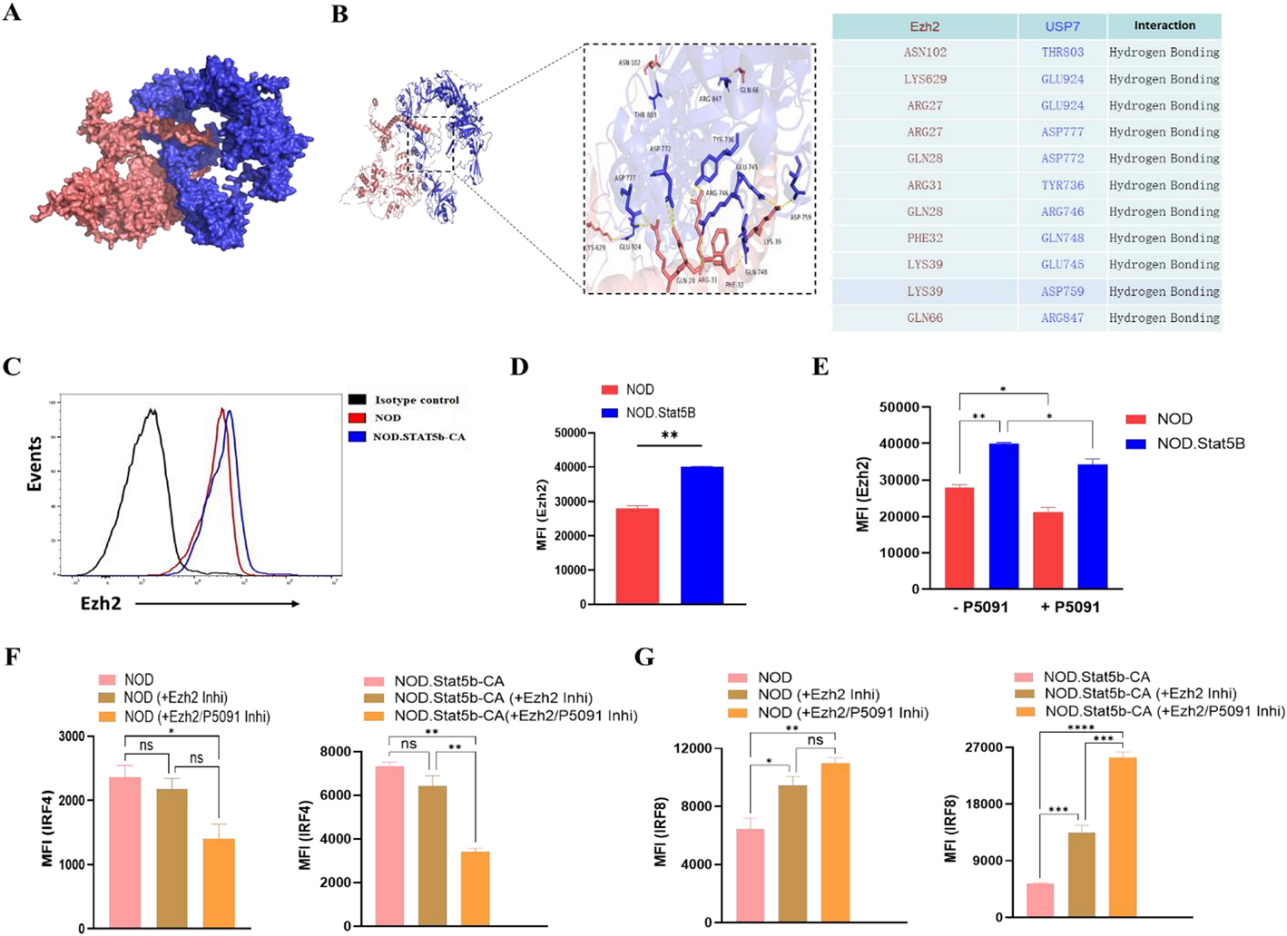
USP7 controls IRF4 and IRF8 expression by regulating Ezh2. **A** Crystal structure of overall protein–protein binding between USP7 and Ezh2. **B** Ribbon representation of the crystal structure of USP7:Ezh2 peptide complex with the USP7 peptide represented by blue color and Ezh2 peptide represented by red color (left). The interactions formed between USP7 and the Ezh2 peptide are shown as yellow dashed lines. The residues involved in the interactions are labeled (right) and presented in table form. **C**–**G** Splenic DCs purified from NOD and NOD.Stat5b-CA mice were incubated with either vehicle (0.1% DMSO) or with the Ezh2 inhibitor GSK343 (3 μM/mL) alone or together with the USP7 inhibitor P5091 (5 μM/mL) for 24 h. Thereafter, cells were washed and then stained with anti-CD11c, anti-Ezh2, anti-IRF4, and anti-IRF8 antibodies and analyzed by flow cytometry. **C**, **D** Histogram profile (left panel) and Mean fluorescence intensity (MFI) quantification (right panel) of the level of Ezh2 expression in CD11c^+^ DCs. Data are shown as the mean ± SEM of three independent experiments. The two-tailed unpaired Student’s *t*-test was used. **P < 0.01. **E** MFI values of Ezh2 in DCs treated with either vehicle or P5091 inhibitor. **F**, **G** MFI values of IRF4 and IRF8 in vehicle-treated DCs or DCs treated with Ezh2 inhibitor alone or together with P5091 inhibitor. Data are shown as the mean ± SEM of three independent experiments. One-way ANOVA followed by Tukey’s post hoc test was used. n.s, not significant; *P < 0.05; **P < 0.01; ***P < 0.001; ****P < 0.0001

Previously, we have found that Stat5b and Ezh2 are recruited to limit IRF8 but not IRF4 transcription in DCs of NOD and NOD.Stat5b-CA mice [14, 39]. Moreover, the above data showed that USP7 regulates both IRF4 and IRF8, raising the question of whether Ezh2 is involved in the regulation of these transcription factors by USP7, which has not yet been investigated. Recent studies showed that USP7 interacts with Ezh2 and promotes its stabilization in cancer cells and that loss of USP7 leads to Ezh2 destabilization [43, 44]. To evaluate the protein–protein interaction, we first mapped BioPlex edges onto three-dimensional structures of USP7 and Ezh2 drawn from the Protein Data Bank (PDB) database (http://huanglab.phys.hust.edu.cn/). As reported before [43, 44], we confirmed a direct interaction and a complex formation between USP7 and Ezh2 (Fig. 6A, B). Next, we examined whether inhibiting USP7 affects Ezh2 expression. Data showed that Ezh2 expression level was elevated in cDCs from NOD.Stat5b-CA mice than in cDCs from NOD mice (Fig. 6C, D) and that the inhibition of USP7 significantly downregulated Ezh2 expression in cDCs from both strains of mice (Fig. 6E). These results indicate that USP7 directly regulates Ezh2 expression in cDCs. To elucidate the role of Ezh2 in IRF4 and IRF8 regulation by USP7, we assessed the impact of Ezh2 and USP7 inhibition on IRF4 and IRF8 expression. As we previously reported [14, 39], Ezh2 inhibition upregulated IRF8 expression but had no effect on IRF4 expression in the cDCs of both mouse strains (Fig. 6F–G). Interestingly, inhibition of both Ezh2 and USP7 resulted in a significant downregulation of IRF4 and an upregulation of IRF8 expression (Fig. 6F–G). These data suggest that USP7 regulates IRF4 expression in an Ezh2-independent manner, whereas IRF8 expression is regulated by a USP7-Ezh2-dependent pathway.

### USP7 inhibition reprograms tolerogenic DCs to promote Th17 while restraining Th2 and Treg differentiation

**Fig. 7:**
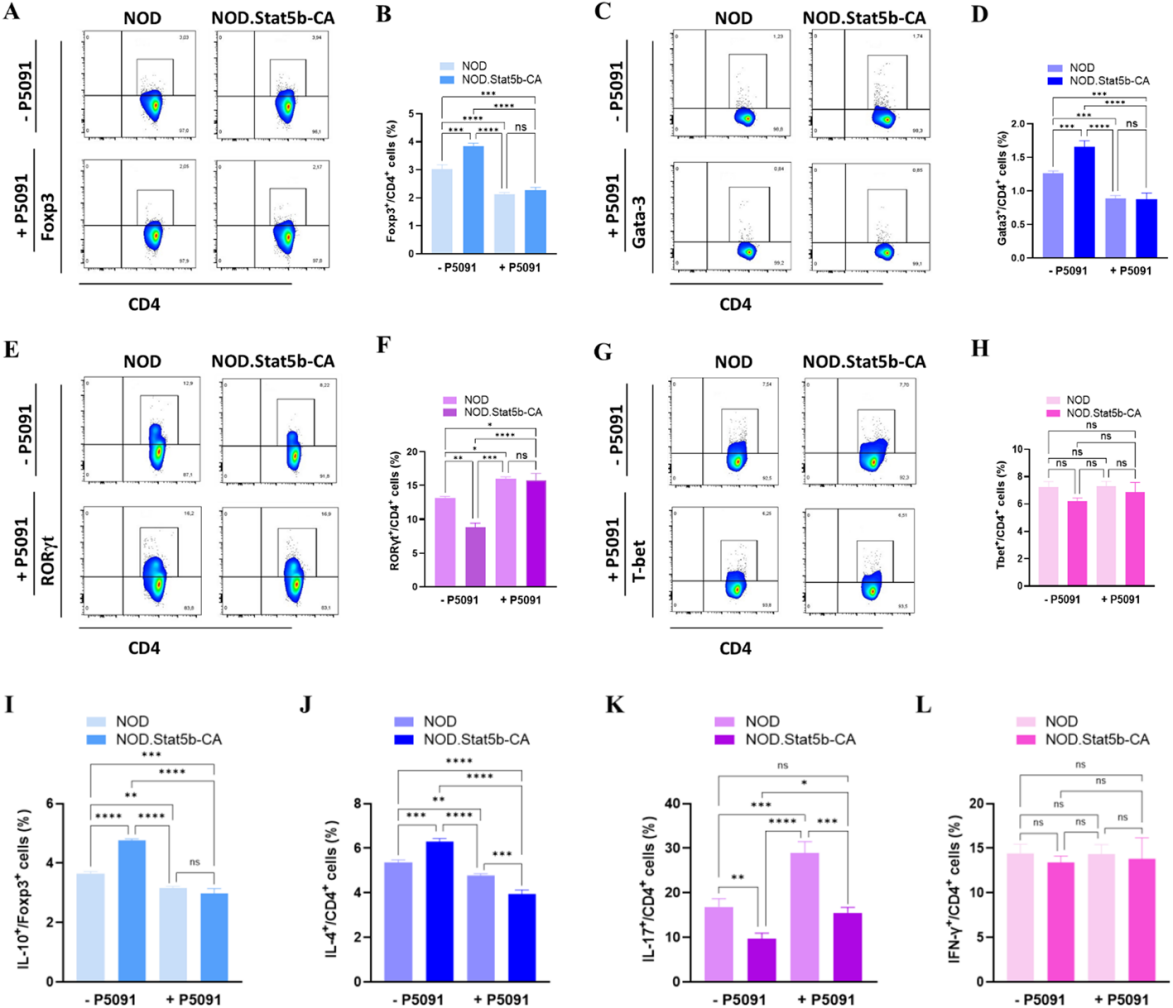
Inhibition of USP7 in tolerogenic Stat5b-CA.DCs reduce Tregs and Th2 responses and promote IL-17-producing Th17 cell subset. Purified splenic DCs from NOD and NOD.Stat5b-CA mice were pre-incubated with (+ PD5091) or without (− PD509) USP7 inhibitor (5 µM/mL) for 24 h and then stimulated with LPS (1 µg/mL) for additional 24 h. Cells were then washed, and 6 × 10^6^ cells were i.v injected into 8–10 weeks old NOD mice. After 7 days, spleen cells were harvested and analyzed for Tregs and Th1/Th2/Th17 cell subsets and their cytokine profiles by FACS. **A**–**H** Representative FACS profile and percentage of CD4^+^Foxp3^+^ Tregs (**A**, **B**), CD4^+^Gata-3^+^ Th2 (C-D), CD4^+^RORγt^+^ Th17 (**E**, **F**) and CD4^+^T-bet^+^ Th1 (**G**, **H**) cells. **I**–**L** Splenic cells of recipient NOD mice were stained with different T cell subsets and cytokine antibodies and analyzed by FACS. Percentages of Foxp3^+^IL-10^+^ (**I**), CD4^+^IL-4^+^ (**J**), CD4^+^IL-17^+^ (**K**), and CD4^+^IFN-γ^+^ (**L**) T cell subsets. In all experiments, 4 mice per group were used, and the results are expressed as mean ± SEM. The significance was calculated using One-way ANOVA with Tukey’s post-test. n.s., not significant; *P < 0.05; **P < 0.01; ***P < 0.001; ****P < 0.0001

Since tolerogenic DCs of NOD.Stat5b-CA promotes Tregs and Th2 over Th1 differentiation [13], we sought to investigate the impact of USP7 inhibition in Stat5b-CA.DCs on T helper cells and Tregs immune response. For this purpose, vehicle- or USP7 inhibitor-treated splenic cDCs of NOD and NOD.Stat5b-CA mice were stimulated with LPS and then injected into prediabetic NOD mice. After 7 days, splenic T cells and cytokine profiles of recipient mice were analyzed by flow cytometry. The results showed that the frequencies and absolute numbers of CD4^+^Foxp3^+^ Tregs (Fig. 7A, B and Supplementary Fig. 3 A) and CD4^+^Gata3^+^ Th2 cells (Fig. 7C, D and Supplementary Fig. 3B) were markedly increased in NOD mice injected with DCs of NOD.Stat5b-CA mice as compared to NOD mice injected with DCs of NOD mice. However, the frequency and the absolute numbers of RORγt^+^ Th17 cells were markedly reduced in NOD mice injected with DCs of NOD.Stat5b-CA in comparison to the mice injected with NOD DCs (Fig. 7E, F and Supplementary Fig. 3 C). Similarly, we found a prominent reduction in the total number of CD4^+^Tbet^+^ Th1 cells between NOD and NOD.Stat5b-CA injected DCs while no significant difference was found in the frequency of CD4^+^Tbet^+^ cells in the spleens of both recipient mice (Fig. 7G, H and Supplementary Fig. 3D). Importantly, the frequencies and absolute number of Tregs and Th2 cells in the spleens of NOD mice transfused with USP7 inhibitor pre-treated DCs from NOD or NOD.Stat5b-CA mice were significantly reduced (Fig. 7A–D and Supplementary Fig. 3 A, B) while their Th17 cell frequency and absolute numbers were significantly increased (Fig. 7E, F and Supplementary Fig. 3 C). Similarly, IL-10-producing Tregs and IL-4-producing Th2 cell frequencies were significantly increased in the spleens of recipient mice transfused with DCs of NOD.Stat5b-CA mice which were decreased in the recipient mice injected with USP7 inhibitor-pre-treated DCs (Fig. 7I, J). The decrease in IL-10-producing Tregs and IL-4-producing Th2 cells was accompanied by an increased IL-17-producing Th17 cell frequency (Fig. 7K), while the frequency of IFNγ-producing Th1 cells remained unaltered (Fig. 7L). Together, these results demonstrate the importance of USP7 in DC tolerogenic function to regulate Tregs, Th2, and Th17 cell immune responses in NOD mice.

### USP7 blockade reduces the tolerogenic functions of Stat5b-CA.DCs, restoring their capacity to induce diabetes development

**Fig. 8:**
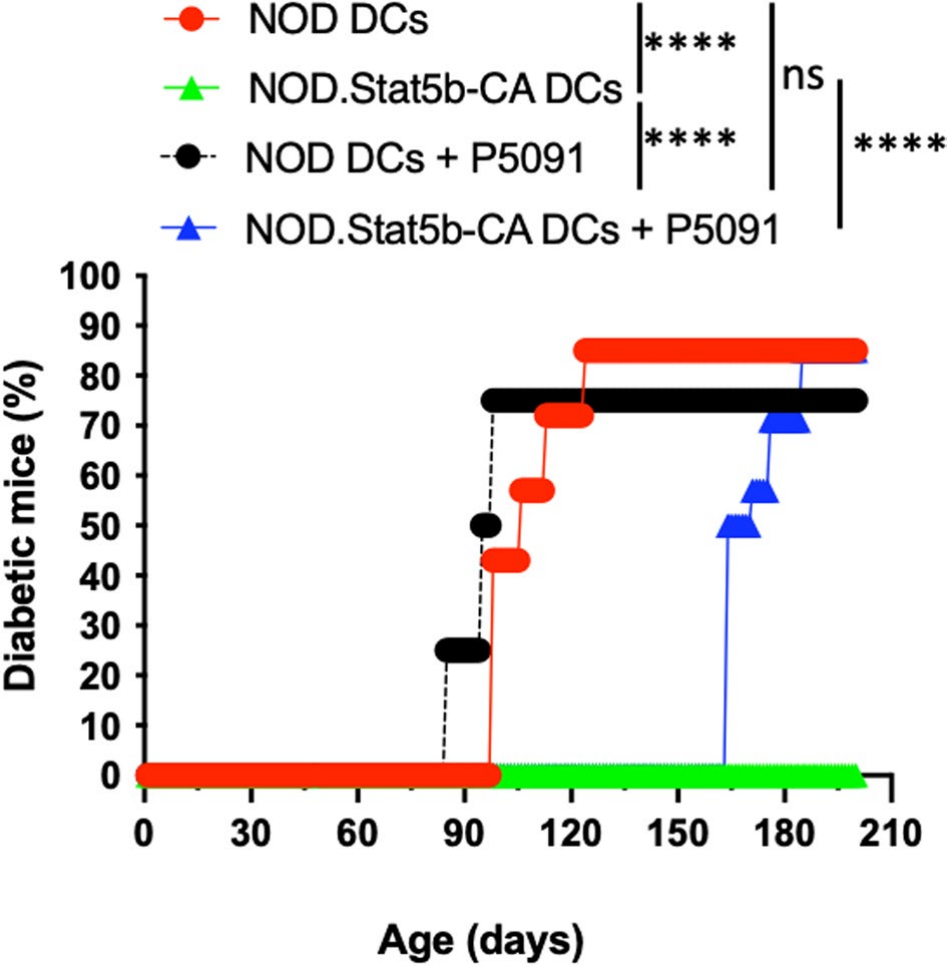
USP7 blockade halts the capacity of tolerogenic Stat5b-CA.DCs to protect NOD mice from diabetes. Purified splenic DC of NOD and NOD.Stat5b-CA mice were treated or not with USP7 inhibitor P5091 (5 µM/mL, for 24 h), stimulated with LPS for another 24 h, and intravenously injected into 3–4 weeks old female NOD mice (7 mice per group, 6 × 10^6^ cells/mouse). Recipient NOD mice were followed for diabetes development until 32 weeks of age. For comparison of diabetes incidence between different groups, the Gehan–Breslow–Wilcoxon test was performed to calculate the significant difference. n.s., not significant; ****P < 0.0001

The above results showed that the tolerogenic function of DCs of NOD.Stat5b-CA mice is lost when treated with USP7 inhibitor, as demonstrated by the promotion of pro-inflammatory Th17 cells and reduction of anti-inflammatory Th2 and Tregs cell differentiation. These findings led us to investigate whether the USP7 blockade was devoid of the capacity of tolerogenic Stat5b-CA.DCs to halt the development of diabetes in NOD mice. To this end, vehicle- or USP7 inhibitor-treated splenic DCs of NOD and NOD.Stat5b-CA mice were stimulated with LPS and intravenously transfused into nondiabetic young NOD mice that were followed for diabetes. Results showed that similar to diabetes incidence in NOD mice in our mouse colony, 80–90% of NOD mice transfused with the vehicle- or USP7 inhibitor-treated NOD DCs become diabetic (Fig. 8). As we previously reported [13], NOD mice transduced with the vehicle-treated DCs of NOD.Stat5b-CA mice were fully protected from diabetes (Fig. 8). Of importance, when treated with USP7 inhibitor, DCs of NOD.Stat5b-CA mice failed to protect NOD recipient mice from developing diabetes (Fig. 8). These data strongly demonstrate that USP7 contributes to maintaining NOD.Stat5b-CA mice DCs tolerogenic capacity to establish immunological tolerance and to prevent diabetes development in NOD mice.

## Discussion

Using a spontaneous model of type 1 diabetes NOD mice, we have reported that splenic DCs expressing an active form of Stat5b transcription factor (Stat5b-CA.DCs) exhibit the signature of tolerogenic DCs such as expressing high levels of PD-L1 and PD-L2, producing a large amount of TGF-β and protecting NOD mice from the development of autoimmune diabetes [13, 14, 39]. In this study, we found that tolerogenic Stat5b-CA.DCs express higher levels of ubiquitin-specific protease USP7 and that treatment of Stat5b-CA.DCs with the USP7 inhibitor P5091 weaken their tolerogenic properties and reprogrammed them to exhibit immunogenic DCs properties. USP7 inhibition in tolerogenic Stat5b-CA.DCs increased their maturation phenotype while reducing PD-L1 and PDL-2 expression and enhancing the production of proinflammatory cytokines TNF-α, IL-1β, and IL-6. Mechanistically, USP7 inhibition reduced Stat5b-CA.DC IRF4 expression in an Ezh2-independent manner while upregulating IRF8 expression via Ezh2 and promoting cDC1 over cDC2 subsets. Moreover, USP7 inhibition increased Stat5b-CA.DC capacity to promote Th17 cells, restrained Th2 and Treg cells in vivo and, therefore, lost their capacity to protect NOD mice from diabetes.

In autoimmunity, prevention, or promotion of peripheral and tissue-specific autoimmune response is influenced by the DC phenotype, subtypes, and functions. In agreement with this conclusion, it has been largely shown that DCs from NOD mice and monocytes-derived DCs from diabetic patients are abnormally activated, produce high amounts of proinflammatory cytokines, and contribute to autoreactive T cell activation and diabetes development [45–47]. Immunosuppressive DCs that are functionally classified as tolerogenic are responsible for the induction and maintenance of self-tolerance. Several studies have demonstrated that in experimental animal models, semimature or alternatively activated DCs can control autoreactive T-cell responses and restore Ag-specific tolerance [48, 49]. We have reported that immunogenic DCs of NOD mice engineered to express active Stat5b are tolerogenic and are successful in treating ongoing diabetes in the preclinical NOD mouse model [13]. Understanding the mechanisms involved in reprogramming immunogenic DCs to tolerogenic DCs is crucial for the design of successful DC therapy to suppress autoimmune diseases.

The ubiquitinating/deubiquitinating system is a crucial regulatory mechanism that cells use to control the breakdown and homeostasis of proteins generated by external stimuli. It is also intimately related to development and the regulation of the inflammatory immune system [50]. Among deubiquitinating enzymes, USP7 is the most widely studied in the context of cancer [51, 52]. Previous studies have pinpointed the important role of USP7 in sustained intra-tumoral highly immunosuppressive Foxp3^+^ Tregs that limit effector T cell antitumor responses [53, 54]. USP7 has been shown to interact with Foxp3 in Tregs, and the knockdown of USP7 hindered Tregs immunosuppressive function [30, 31]. On the contrary, USP7 expression decreased polyubiquitination of Foxp3 and enhanced Foxp3 expression (30). In contrast, inhibition, or knockdown of USP7, decreased Foxp3 expression and Tregs suppressive function [30, 55]. However, the role of USP7 in DCs is largely unknown. Up to date, only one report examined the role of USP7 in pDCs and explored whether USP7 affects pDCs maturation and function in multiple myeloma [33]. They reported that treatment of MM patient’s pDCs with USP7 inhibitor (XL177 A) upregulated the expression of CD83, CD86, and HAL-DR MHC class II and increased the release of INF-α. They also showed that USP7 blockade induced pDCs activation and restored MM-specific cytotoxic CD8^+^ T lymphocytes and NK cell-mediated activity. Our study revealed for the first time a higher expression of USP7 in tolerogenic Stat5b-CA.DCs than in immunogenic cDCs in the autoimmune setting. In agreement with this report [33], we found that USP7 inhibition increased levels of maturation markers CD80, CD86, CD40, and MHC class II expression, indicating that USP7 blocked induced DC overactivation. Beyond DC maturation, inhibition of USP7 promoted tolerogenic DC production of proinflammatory cytokines (TNF-α, IL-1β, and IL-6) while repressing IL-10 and TGF-β production, indicating tolerogenic Stat5b-CA.DCs acquired an immunogenic phenotype. These findings suggest an immunomodulatory role of USP7 in tolerogenic DCs function.

We further showed that tolerogenic Stat5b-CA.DCs expressed higher levels of PD-L1 and PD-L2 than immunogenic DCs of NOD mice, and that USP7 inhibition downregulated their PD-L1 and PD-L2 expression levels. In agreement with our findings, it has been shown that USP7 is overexpressed in gastric tumors and that USP7 directly binds with PDL-1 to induce its deubiquitination and stabilization [56] and suggested that USP7 abrogation could be used as another approach to downregulate PD-L1 and render gastric cancer cells sensitive to the killing by T cells. On the contrary, another study found that USP7 inhibition elevated the PD-L1 expression in lung cancer cells [57], suggesting that the regulation of PD-L1 expression by USP7 is multifaceted and may be context-dependent.

Different subsets of DCs are specialized for eliciting specific elements of the immune response. DCs that induce Th1 and Th17 or Th2 and Treg responses have been designated as cDC1 or cDC2 cells, respectively [58, 59]. Along with USP7 immunomodulatory capability, our data showed that low USP7 expressing cDCs of NOD mice have more cDC1 than cDC2 subsets, whereas high USP7 expressing Stat5b-CA.DCs contain more cDC2 than cDC1 cells, and that USP7 inhibition augments cDC1 over DC2 subsets in NOD and NOD.Stat5b-CA mice. Interestingly, USP7 is involved in M1 and M2 macrophage homeostasis regulation. Similar to our finding, USP7 has been shown to be highly expressed in M2 but not in M1 macrophages [57]. Particularly, inhibition of USP7 was shown to reprogram tumor-associated macrophages to M1 macrophages through p38 MAPK pathway activation, thereby enhancing anti-tumor immune responses [57]. Similarly, another study reported that USP7 polarizes anti-inflammatory M2 macrophage phenotype to pro-inflammatory M1 [60].

cDC1 s and cDC2 s express distinct transcriptional programs [61]. IRF4 and IRF8 were found important for cDC development. IRF8 is expressed at all stages of cDC1 development, whereas the development of cDC2 s is governed by IRF4 [8, 62–66]. Moreover, DC-specific deletion of IRF4 resulted in an increased cDC1/cDC2 ratio [8] whereas IRF8^−/−^ mice lack cDC1 development [67]. Our results showed higher expression of IRF4 than IRF8 in tolerogenic cDCs of NOD.Stat5b-CA mice and higher expression of IRF8 than IRF4 in NOD mice, which explains the higher percentage of cDC2 than cDC1 in NOD.Stat5b-CA mice. USP7 blockade downregulated IRF4 and upregulated IRF8 expression in DCs of NOD.Stat5b-CA and NOD mice, which resulted in fostering cDC1 over cDC2 subsets. This supports the notion of USP7-dependent IRF4 and IRF8 regulation and cDC1 and cDC2 development. The finding that cDC1 cells are present at early islet inflammation in NOD mice and that NOD with less cDC1 do not develop diabetes [68, 69], suggests that cDC1 contributes to the initiation and development of diabetes in NOD mice. In agreement with this suggestion, our data showed that transfer of NOD.Stat5b-CA DCs that contain more cDC2 subsets protect NOD mice from diabetes, whereas transfer of DCs of NOD mice that contain more cDC1 do not protect NOD mice from diabetes. We also found that following the USP7 blockade, DCs of NOD.Stat5b-CA mice lost their ability to protect NOD mice from diabetes, indicating the important immunoregulatory role of USP7 in the process of autoimmune tolerance mediated by tolerogenic Stat5b-CA.DCs.

We have also shed light on the mechanisms by which USP7 controls IRF4 and IRF8 expression in cDCs in the context of autoimmunity. Our previous work showed that Ezh2 is involved in IRF8 but not in IRF4 regulation in tolerogenic Stat5b-CA.DCs [14], here we found that the inhibition of USP7 and Ezh2 in cDCs affect both IRF4 and IRF8 expression, suggesting the involvement of USP7-Ezh2 pathway in regulating IRF8 expression whereas IRF4 is regulated by USP7-Ezh2-independent pathway. Thus, our study uncovers for the first time a dual regulatory role of USP7 in controlling IRF4 and IRF8 expression in DCs in Ezh2-dependent and -independent pathways, respectively. The finding that USP7 interacts with Ezh2 is consistent with previous reports where USP7 was found to interact with and stabilize Ezh2 and that loss of USP7 reduced Ezh2 expression in cancer cells [43, 44]. Our finding highlights the complexity of transcriptional regulation in DCs and adds another layer of complexity by identifying that USP7 regulates IRF8 through Ezh2 but IRF4 in an Ezh2-independent manner.

T cells that have been educated by DCs are able to orchestrate immune responses and contribute to either the development of autoimmunity or the maintenance of immune tolerance. It is well established that Th1, Th17, and the defect in Th2 and Tregs contribute to the development of diabetes in NOD mice and humans [70–74]. Earlier studies in NOD mice have shown that the immune tolerance induced by DCs involves diverse cellular mechanisms, such as deletion of T cells [9, 75, 76], immune deviation toward Th2 response, and/or Treg induction/expansion that can induce dominant tolerance [77]. Our in vivo data demonstrated that high USP7 expression by tolerogenic DCs of NOD.Stat5b-CA mice is necessary for the induction and maintenance of Th2 response and Tregs differentiation and that USP7 inhibition reprograms tolerogenic DCs to promote more Th17 while restraining Th2 and Tregs differentiation. This is consistent with our previous finding showing that tolerogenic cDC of NOD.Stat5b-CA promoted Th2 and Tregs responses, while downregulating cytokines involved in Th1 and Th17 immune responses induction [13].

This study has some limitations and raises several outstanding questions. Even though we have found that USP7 differentially regulates IRF4 and IRF8 transcription factors in Ezh2 independent and Ezh2 dependent pathways in tolerogenic versus immunogenic DCs, we have not determined how USP7 regulates Ezh2, IRF4, and IRF8 such as via ubiquitination and proteasome degradation and/or phosphorylation. Furthermore, USP7 is known to have several target proteins particularly those involved in IRF4 regulation; therefore, performing a transcriptomic analysis of immunogenic and tolerogenic DCs in the absence or presence of USP7 inhibitor may reveal new targets that play a crucial role in tolerogenic versus immunogenic DC function and cDC1/cDC2 differentiation in the context of autoimmunity.

## Conclusions

This study provides the first experimental evidence that the USP7/Ezh2 axis plays a crucial role in the tolerogenic function of DCs in the context of autoimmune diabetes. By regulating DC maturation, cytokine production, and DC subset differentiation, USP7 controls the balance between immune tolerance and inflammation. USP7 inhibition disrupts this balance by promoting a pro-inflammatory state and enhancing the immunogenicity of DCs. These findings highlight the USP7/Ezh2 axis as a potential therapeutic target for modulating immune responses in T1D and may offer a new avenue for preventing or treating autoimmune diabetes.

## Supplementary Information

Supplementary Material 1.

## Data Availability

All data generated or analyzed during this study are included in this study and its supplementary information files. The datasets used and/or analyzed during the current study are also available from the corresponding author upon reasonable request.

## Abbreviations

APC: Antigen-presenting cell
cDC: Conventional dendritic cell
cDC1: Conventional dendritic cell type 1
cDC2: Conventional dendritic cell type 2
DC: Dendritic cell
DUB: Deubiquitinase
Ezh2: Enhancer of zeste homolog 2
FACS: Fluorescence-activated cell sorting
FOXP3: Forkhead box P3
IL: Interleukin
IRF: Interferon regulatory factor
LPS: Lipopolysaccharide
MHC: Major histocompatibility complex
MM: Multiple myeloma
NOD: Non-obese diabetic
PD-L1/2: Programmed death-ligand 1/2
RPMI: Roswell Park Memorial Institute (medium)
SEM: Standard error of the mean
STAT5b: Signal transducer and activator of transcription 5b
T1D: Type 1 diabetes
TGF-β: Transforming growth factor-beta
Th: T-helper cell
Treg: Regulatory T cell
TNF-α: Tumor necrosis factor-alpha
USP7: Ubiquitin-specific protease 7

## References

[1] Khosravi-Maharlooei M, Madley R, Borsotti C, Ferreira LMR, Sharp RC, Brehm MA, et al. Modeling human T1D-associated autoimmune processes. Mol Metab. 2022;56: 101417. 34902607 10.1016/j.molmet.2021.101417 PMC8739876

[2] Toren E, Burnette KS, Banerjee RR, Hunter CS, Tse HM. Partners in crime: beta-cells and autoimmune responses complicit in type 1 diabetes pathogenesis. Front Immunol. 2021;12. 10.3389/fimmu.2021.756548 PMC8529969 34691077

[3] Amodio G, Gregori S. Dendritic cells a double-edge sword in autoimmune responses. Front Immunol. 2012;3:233. 22876246 10.3389/fimmu.2012.00233 PMC3410601

[4] Liu J, Cao X. Regulatory dendritic cells in autoimmunity: a comprehensive review. J Autoimmun. 2015;63:1–12. 26255250 10.1016/j.jaut.2015.07.011

[5] Passeri L, Marta F, Bassi V, Gregori S. Tolerogenic dendritic cell-based approaches in autoimmunity. Int J Mol Sci. 2021;22(16):8415. 34445143 10.3390/ijms22168415 PMC8395087

[6] Zirpel H, Roep BO. Islet-resident dendritic cells and macrophages in type 1 diabetes: in search of bigfoot’s print. Front Endocrinol. 2021;12. 10.3389/fendo.2021.666795 PMC8072455 33912139

[7] Liu J, Zhang X, Cheng Y, Cao X. Dendritic cell migration in inflammation and immunity. Cell Mol Immunol. 2021;18(11):2461–71. 34302064 10.1038/s41423-021-00726-4 PMC8298985

[8] Iberg CA, Jones A, Hawiger D. Dendritic cells as inducers of peripheral tolerance. Trends Immunol. 2017;38(11):793–804. 28826942 10.1016/j.it.2017.07.007 PMC5669994

[9] Price JD, Tarbell KV. The role of dendritic cell subsets and innate immunity in the pathogenesis of type 1 diabetes and other autoimmune diseases. Front Immunol. 2015;6. 10.3389/fimmu.2015.00288 PMC4466467 26124756

[10] Welzen-Coppens JM, van Helden-Meeuwsen CG, Drexhage HA, Versnel MA. Abnormalities of dendritic cell precursors in the pancreas of the NOD mouse model of diabetes. Eur J Immunol. 2012;42(1):186–94. 22002898 10.1002/eji.201141770

[11] Vasquez AC, Feili-Hariri M, Tan RJ, Morel PA. Qualitative and quantitative abnormalities in splenic dendritic cell populations in NOD mice. Clin Exp Immunol. 2004;135(2):209–18. 14738447 10.1111/j.1365-2249.2003.02359.x PMC1808940

[12] Hansen L, Schmidt-Christensen A, Gupta S, Fransén-Pettersson N, Hannibal TD, Reizis B, et al. E2–2 dependent plasmacytoid dendritic cells control autoimmune diabetes. PLoS ONE. 2015;10(12): e0144090. 26624013 10.1371/journal.pone.0144090 PMC4666626

[13] Zerif E, Maalem A, Gaudreau S, Guindi C, Ramzan M, Véroneau S, et al. Constitutively active Stat5b signaling confers tolerogenic functions to dendritic cells of NOD mice and halts diabetes progression. J Autoimmun. 2017;76:63–74. 27634616 10.1016/j.jaut.2016.09.001

[14] Zerif E, Khan FU, Raki AA, Lullier V, Gris D, Dupuis G, et al. Elucidating the role of Ezh2 in tolerogenic function of NOD bone marrow-derived dendritic cells expressing constitutively active Stat5b. Int J Mol Sci. 2020;21(18):6453. 32899608 10.3390/ijms21186453 PMC7554732

[15] Khan FU, Khongorzul P, Raki AA, Rajasekaran A, Gris D, Amrani A. Dendritic cells and their immunotherapeutic potential for treating type 1 diabetes. Int J Mol Sci. 2022;23(9). 10.3390/ijms23094885 PMC9099521 35563276

[16] Jongbloed SL, Kassianos AJ, McDonald KJ, Clark GJ, Ju X, Angel CE, et al. Human CD141+ (BDCA-3)+ dendritic cells (DCs) represent a unique myeloid DC subset that cross-presents necrotic cell antigens. J Exp Med. 2010;207(6):1247–60. 20479116 10.1084/jem.20092140 PMC2882828

[17] Mortha A, Burrows K. Cytokine networks between innate lymphoid cells and myeloid cells. Front Immunol. 2018;9(191). 10.3389/fimmu.2018.00191 PMC5808287 29467768

[18] Segura E, Valladeau-Guilemond J, Donnadieu MH, Sastre-Garau X, Soumelis V, Amigorena S. Characterization of resident and migratory dendritic cells in human lymph nodes. J Exp Med. 2012;209(4):653–60. 22430490 10.1084/jem.20111457 PMC3328358

[19] Leal Rojas IM, Mok W-H, Pearson FE, Minoda Y, Kenna TJ, Barnard RT, et al. Human blood CD1c(+) dendritic cells promote Th1 and Th17 effector function in memory CD4(+) T cells. Front Immunol. 2017;8:971. 28878767 10.3389/fimmu.2017.00971 PMC5572390

[20] Russler-Germain EV, Yi J, Young S, Nutsch K, Wong HS, Ai TL, et al. Gut Helicobacter presentation by multiple dendritic cell subsets enables context-specific regulatory T cell generation. Elife. 2021;10: e54792. 33533717 10.7554/eLife.54792 PMC7877908

[21] Kim RQ, Geurink PP, Mulder MPC, Fish A, Ekkebus R, El Oualid F, et al. Kinetic analysis of multistep USP7 mechanism shows critical role for target protein in activity. Nat Commun. 2019;10(1):231. 30651545 10.1038/s41467-018-08231-5 PMC6335408

[22] Zhang C, Lu J, Zhang QW, Zhao W, Guo JH, Liu SL, et al. USP7 promotes cell proliferation through the stabilization of Ki-67 protein in non-small cell lung cancer cells. Int J Biochem Cell Biol. 2016;79:209–21. 27590858 10.1016/j.biocel.2016.08.025

[23] Li J, Dai Y, Ge H, Guo S, Zhang W, Wang Y, et al. The deubiquitinase USP7 promotes HNSCC progression via deubiquitinating and stabilizing TAZ. Cell Death Dis. 2022;13(8):677. 35931679 10.1038/s41419-022-05113-z PMC9356134

[24] Wang Z, Kang W, You Y, Pang J, Ren H, Suo Z, et al. USP7: novel drug target in cancer therapy. Front Pharmacol. 2019;10:427. 31114498 10.3389/fphar.2019.00427 PMC6502913

[25] Inoue D, Nishimura K, Kozuka-Hata H, Oyama M, Kitamura T. The stability of epigenetic factor ASXL1 is regulated through ubiquitination and USP7-mediated deubiquitination. Leukemia. 2015;29(11):2257–60. 25836587 10.1038/leu.2015.90

[26] Valles GJ, Bezsonova I, Woodgate R, Ashton NW. USP7 is a master regulator of genome stability. Front Cell Dev Biol. 2020;8. 10.3389/fcell.2020.00717 PMC7419626 32850836

[27] Su D, Ma S, Shan L, Wang Y, Wang Y, Cao C, et al. Ubiquitin-specific protease 7 sustains DNA damage response and promotes cervical carcinogenesis. J Clin Investig. 2018;128(10):4280–96. 30179224 10.1172/JCI120518 PMC6159995

[28] Qiao H, Tian Y, Huo Y, Man H-Y. Role of the DUB enzyme USP7 in dendritic arborization, neuronal migration, and autistic-like behaviors in mice. iScience. 2022;25(7): 104595. 35800757 10.1016/j.isci.2022.104595 PMC9253496

[29] Bhattacharya S, Chakraborty D, Basu M, Ghosh MK. Emerging insights into HAUSP (USP7) in physiology, cancer and other diseases. Signal Transduct Target Ther. 2018;3(1):17. 29967688 10.1038/s41392-018-0012-y PMC6023882

[30] van Loosdregt J, Fleskens V, Fu J, Brenkman AB, Bekker CP, Pals CE, et al. Stabilization of the transcription factor Foxp3 by the deubiquitinase USP7 increases Treg-cell-suppressive capacity. Immunity. 2013;39(2):259–71. 23973222 10.1016/j.immuni.2013.05.018 PMC4133784

[31] Wang L, Kumar S, Dahiya S, Wang F, Wu J, Newick K, et al. Ubiquitin-specific protease-7 inhibition impairs Tip60-dependent Foxp3+ T-regulatory cell function and promotes antitumor immunity. EBioMedicine. 2016;13:99–112. 27769803 10.1016/j.ebiom.2016.10.018 PMC5264272

[32] Okeke EB, Uzonna JE. The pivotal role of regulatory T cells in the regulation of innate immune cells. Front Immunol. 2019;10. 10.3389/fimmu.2019.00680 PMC6465517 31024539

[33] Ray A, Du T, Song Y, Buhrlage SJ, Chauhan D, Anderson K. Blockade of deubiquitylating enzyme USP7 in plasmacytoid dendritic cells stimulates anti-myeloma immunity. Blood. 2020;136:43.

[34] Chauhan D, Tian Z, Nicholson B, Kumar KG, Zhou B, Carrasco R, et al. A small molecule inhibitor of ubiquitin-specific protease-7 induces apoptosis in multiple myeloma cells and overcomes bortezomib resistance. Cancer Cell. 2012;22(3):345–58. 22975377 10.1016/j.ccr.2012.08.007 PMC3478134

[35] Fan YH, Cheng J, Vasudevan SA, Dou J, Zhang H, Patel RH, et al. USP7 inhibitor P22077 inhibits neuroblastoma growth via inducing p53-mediated apoptosis. Cell Death Dis. 2013;4(10): e867. 24136231 10.1038/cddis.2013.400 PMC3920959

[36] Godoy GJ, Olivera C, Paira DA, Salazar FC, Ana Y, Stempin CC, et al. T regulatory cells from non-obese diabetic mice show low responsiveness to IL-2 stimulation and exhibit differential expression of anergy-related and ubiquitination factors. Front Immunol. 2019;10. 10.3389/fimmu.2019.02665 PMC6886461 31824482

[37] Pozhidaeva A, Valles G, Wang F, Wu J, Sterner DE, Nguyen P, et al. USP7-specific inhibitors target and modify the enzyme’s active site via distinct chemical mechanisms. Cell Chem Biol. 2017;24(12):1501-12.e5. 29056420 10.1016/j.chembiol.2017.09.004

[38] Cartel M, Mouchel P-L, Gotanègre M, David L, Bertoli S, Mansat-De Mas V, et al. Inhibition of ubiquitin-specific protease 7 sensitizes acute myeloid leukemia to chemotherapy. Leukemia. 2021;35(2):417–32. 32447346 10.1038/s41375-020-0878-x PMC7245510

[39] Ullah Khan F, Khongorzul P, Gris D, Amrani A. Stat5b/Ezh2 axis governs high PD-L1 expressing tolerogenic dendritic cell subset in autoimmune diabetes. Int Immunopharmacol. 2024;133: 112166. 38678673 10.1016/j.intimp.2024.112166

[40] Anderson DA 3rd, Murphy KM, Briseno CG. Development, diversity, and function of dendritic cells in mouse and human. Cold Spring Harb Perspect Biol. 2018;10(11): a028613. 28963110 10.1101/cshperspect.a028613 PMC6211386

[41] Nutt SL, Chopin M. Transcriptional networks driving dendritic cell differentiation and function. Immunity. 2020;52(6):942–56. 32553180 10.1016/j.immuni.2020.05.005

[42] Zhang S, Audiger C, Chopin M, Nutt SL. Transcriptional regulation of dendritic cell development and function. Front Immunol. 2023;14:1182553. 37520521 10.3389/fimmu.2023.1182553 PMC10382230

[43] Gagarina V, Bojagora A, Lacdao IK, Luthra N, Pfoh R, Mohseni S, et al. Structural basis of the interaction between ubiquitin specific protease 7 and enhancer of Zeste Homolog 2. J Mol Biol. 2020;432(4):897–912. 31866294 10.1016/j.jmb.2019.12.026

[44] Su D, Wang W, Hou Y, Wang L, Yi X, Cao C, et al. Bimodal regulation of the PRC2 complex by USP7 underlies tumorigenesis. Nucleic Acids Res. 2021;49(8):4421–40. 33849069 10.1093/nar/gkab209 PMC8096222

[45] Feili-Hariri M, Morel PA. Phenotypic and functional characteristics of BM-derived DC from NOD and non-diabetes-prone strains. Clin Immunol. 2001;98(1):133–42. 11141336 10.1006/clim.2000.4959

[46] Marleau AM, Singh B. Myeloid dendritic cells in non-obese diabetic mice have elevated costimulatory and T helper-1-inducing abilities. J Autoimmun. 2002;19(1–2):23–35. 12367556 10.1006/jaut.2002.0597

[47] Steptoe RJ, Ritchie JM, Harrison LC. Increased generation of dendritic cells from myeloid progenitors in autoimmune-prone nonobese diabetic mice. J Immunol. 2002;168(10):5032–41. 11994455 10.4049/jimmunol.168.10.5032

[48] Fucikova J, Palova-Jelinkova L, Bartunkova J, Spisek R. Induction of tolerance and immunity by dendritic cells: mechanisms and clinical applications. Front Immunol. 2019;10:2393. 31736936 10.3389/fimmu.2019.02393 PMC6830192

[49] Morelli AE, Thomson AW. Tolerogenic dendritic cells and the quest for transplant tolerance. Nat Rev Immunol. 2007;7(8):610–21. 17627284 10.1038/nri2132

[50] Fraile JM, Quesada V, Rodriguez D, Freije JM, Lopez-Otin C. Deubiquitinases in cancer: new functions and therapeutic options. Oncogene. 2012;31(19):2373–88. 21996736 10.1038/onc.2011.443

[51] Bhattacharya S, Chakraborty D, Basu M, Ghosh MK. Emerging insights into HAUSP (USP7) in physiology, cancer and other diseases. Signal Transduct Target Ther. 2018;3:17. 29967688 10.1038/s41392-018-0012-y PMC6023882

[52] Harrigan JA, Jacq X, Martin NM, Jackson SP. Deubiquitylating enzymes and drug discovery: emerging opportunities. Nat Rev Drug Discov. 2018;17(1):57–78. 28959952 10.1038/nrd.2017.152 PMC7097658

[53] deLeeuw RJ, Kost SE, Kakal JA, Nelson BH. The prognostic value of FoxP3+ tumor-infiltrating lymphocytes in cancer: a critical review of the literature. Clin Cancer Res. 2012;18(11):3022–9. 22510350 10.1158/1078-0432.CCR-11-3216

[54] Shang B, Liu Y, Jiang SJ, Liu Y. Prognostic value of tumor-infiltrating FoxP3+ regulatory T cells in cancers: a systematic review and meta-analysis. Sci Rep. 2015;5:15179. 26462617 10.1038/srep15179 PMC4604472

[55] Wang F, Wang L, Wu J, Sokirniy I, Nguyen P, Bregnard T, et al. Active site-targeted covalent irreversible inhibitors of USP7 impair the functions of Foxp3+ T-regulatory cells by promoting ubiquitination of Tip60. PLoS ONE. 2017;12(12): e0189744. 29236775 10.1371/journal.pone.0189744 PMC5728538

[56] Wang Z, Kang W, Li O, Qi F, Wang J, You Y, et al. Abrogation of USP7 is an alternative strategy to downregulate PD-L1 and sensitize gastric cancer cells to T cells killing. Acta Pharm Sin B. 2021;11(3):694–707. 33777676 10.1016/j.apsb.2020.11.005 PMC7982505

[57] Dai X, Lu L, Deng S, Meng J, Wan C, Huang J, et al. USP7 targeting modulates anti-tumor immune response by reprogramming tumor-associated macrophages in lung cancer. Theranostics. 2020;10(20):9332–47. 32802195 10.7150/thno.47137 PMC7415808

[58] Starosz A, Jamiolkowska-Sztabkowska M, Glowinska-Olszewska B, Moniuszko M, Bossowski A, Grubczak K. Immunological balance between Treg and Th17 lymphocytes as a key element of type 1 diabetes progression in children. Front Immunol. 2022;13: 958430. 36091019 10.3389/fimmu.2022.958430 PMC9449530

[59] Viisanen T, Gazali AM, Ihantola EL, Ekman I, Nanto-Salonen K, Veijola R, et al. FOXP3+ regulatory T cell compartment is altered in children with newly diagnosed type 1 diabetes but not in autoantibody-positive at-risk children. Front Immunol. 2019;10:19. 30723474 10.3389/fimmu.2019.00019 PMC6349758

[60] Wu J, Kumar S, Wang F, Wang H, Chen L, Arsenault P, et al. Chemical approaches to intervening in ubiquitin specific protease 7 (USP7) function for oncology and immune oncology therapies. J Med Chem. 2018;61(2):422–43. 28768102 10.1021/acs.jmedchem.7b00498

[61] Miller JC, Brown BD, Shay T, Gautier EL, Jojic V, Cohain A, et al. Deciphering the transcriptional network of the dendritic cell lineage. Nat Immunol. 2012;13(9):888–99. 22797772 10.1038/ni.2370 PMC3985403

[62] Bajana S, Turner S, Paul J, Ainsua-Enrich E, Kovats S. IRF4 and IRF8 act in CD11c+ cells to regulate terminal differentiation of lung tissue dendritic cells. J Immunol. 2016;196(4):1666–77. 26746189 10.4049/jimmunol.1501870 PMC4744567

[63] Durai V, Murphy KM. Functions of murine dendritic cells. Immunity. 2016;45(4):719–36. 27760337 10.1016/j.immuni.2016.10.010 PMC5145312

[64] Guilliams M, Dutertre CA, Scott CL, McGovern N, Sichien D, Chakarov S, et al. Unsupervised high-dimensional analysis aligns dendritic cells across tissues and species. Immunity. 2016;45(3):669–84. 27637149 10.1016/j.immuni.2016.08.015 PMC5040826

[65] Sichien D, Scott CL, Martens L, Vanderkerken M, Van Gassen S, Plantinga M, et al. IRF8 transcription factor controls survival and function of terminally differentiated conventional and plasmacytoid dendritic cells, respectively. Immunity. 2016;45(3):626–40. 27637148 10.1016/j.immuni.2016.08.013

[66] Tailor P, Tamura T, Morse HC 3rd, Ozato K. The BXH2 mutation in IRF8 differentially impairs dendritic cell subset development in the mouse. Blood. 2008;111(4):1942–5. 18055870 10.1182/blood-2007-07-100750 PMC2234043

[67] Schiavoni G, Mattei F, Sestili P, Borghi P, Venditti M, Morse HC 3rd, et al. ICSBP is essential for the development of mouse type I interferon-producing cells and for the generation and activation of CD8alpha(+) dendritic cells. J Exp Med. 2002;196(11):1415–25. 12461077 10.1084/jem.20021263 PMC2194263

[68] Ferris ST, Carrero JA, Mohan JF, Calderon B, Murphy KM, Unanue ER. A minor subset of Batf3-dependent antigen-presenting cells in islets of Langerhans is essential for the development of autoimmune diabetes. Immunity. 2014;41(4):657–69. 25367577 10.1016/j.immuni.2014.09.012 PMC4220295

[69] Hotta-Iwamura C, Tarbell KV. Type 1 diabetes genetic susceptibility and dendritic cell function: potential targets for treatment. J Leukoc Biol. 2016;100(1):65–80. 26792821 10.1189/jlb.3MR1115-500R PMC4946618

[70] Besin G, Gaudreau S, Menard M, Guindi C, Dupuis G, Amrani A. Thymic stromal lymphopoietin and thymic stromal lymphopoietin-conditioned dendritic cells induce regulatory T-cell differentiation and protection of NOD mice against diabetes. Diabetes. 2008;57(8):2107–17. 18477807 10.2337/db08-0171 PMC2494678

[71] Bettini M, Bettini ML. Function, failure, and the future potential of tregs in type 1 diabetes. Diabetes. 2021;70(6):1211–9. 34016597 10.2337/dbi18-0058 PMC8275894

[72] Driver JP, Serreze DV, Chen YG. Mouse models for the study of autoimmune type 1 diabetes: a NOD to similarities and differences to human disease. Semin Immunopathol. 2011;33(1):67–87. 20424843 10.1007/s00281-010-0204-1

[73] Ferraro A, Socci C, Stabilini A, Valle A, Monti P, Piemonti L, et al. Expansion of Th17 cells and functional defects in T regulatory cells are key features of the pancreatic lymph nodes in patients with type 1 diabetes. Diabetes. 2011;60(11):2903–13. 21896932 10.2337/db11-0090 PMC3198077

[74] Lee MH, Lee WH, Todorov I, Liu CP. CD4+ CD25+ regulatory T cells prevent type 1 diabetes preceded by dendritic cell-dominant invasive insulitis by affecting chemotaxis and local invasiveness of dendritic cells. J Immunol. 2010;185(4):2493–501. 20639483 10.4049/jimmunol.1001036

[75] Mukhopadhaya A, Hanafusa T, Jarchum I, Chen YG, Iwai Y, Serreze DV, et al. Selective delivery of beta cell antigen to dendritic cells in vivo leads to deletion and tolerance of autoreactive CD8+ T cells in NOD mice. Proc Natl Acad Sci USA. 2008;105(17):6374–9. 18430797 10.1073/pnas.0802644105 PMC2359791

[76] Price JD, Hotta-Iwamura C, Zhao Y, Beauchamp NM, Tarbell KV. DCIR2+ cDC2 DCs and Zbtb32 restore CD4+ T-cell tolerance and inhibit diabetes. Diabetes. 2015;64(10):3521–31. 26070317 10.2337/db14-1880 PMC4587633

[77] Tarbell KV, Petit L, Zuo X, Toy P, Luo X, Mqadmi A, et al. Dendritic cell-expanded, islet-specific CD4+ CD25+ CD62L+ regulatory T cells restore normoglycemia in diabetic NOD mice. J Exp Med. 2007;204(1):191–201. 17210729 10.1084/jem.20061631 PMC2118426

