# Supplementary material for: Role of USP7 in the regulation of tolerogenic dendritic cell function in type 1 diabetes
Source: Cell Mol Biol Lett. 2025 Apr 17;30:47. doi: 10.1186/s11658-025-00727-5 (PMC12004606; doi:10.1186/s11658-025-00727-5)
Supplement: Supplementary file 1 — Supplementary Material 1. [file 11658_2025_727_MOESM1_ESM.docx]

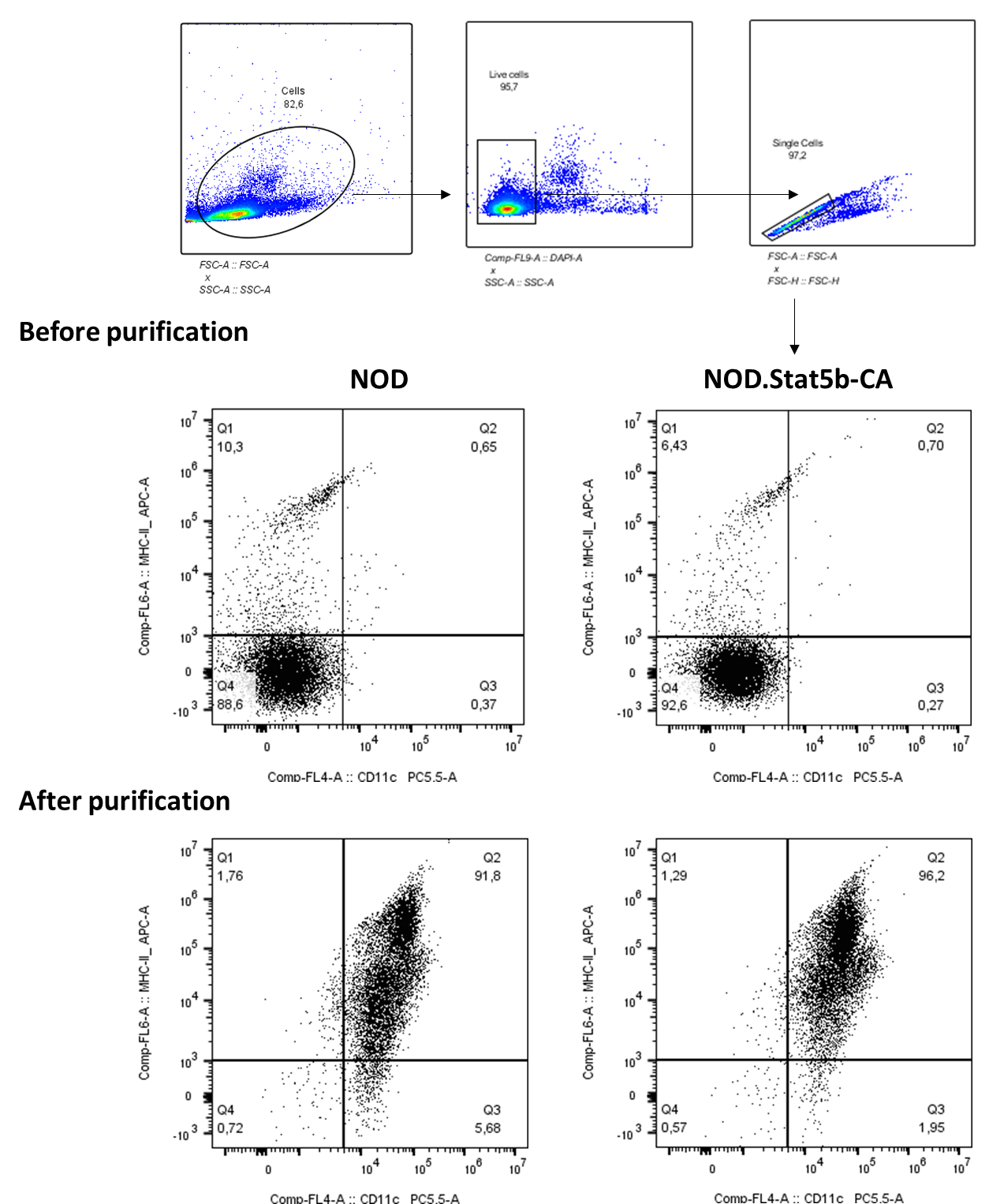


**Supplementary figure 1. Enrichment of dendritic cells from fresh spleens of NOD and NOD.Stat5b-CA mice.** CD11c^+^ dendritic cells were purified from the mouse spleens using CD11c MicroBeads UltraPure isolation kit from Miltenyi Biotech. The purity of the isolated cells was assessed by staining with CD11c-Percp-CY5.5 and MHC-II-APC fluorescent antibodies, followed by flow cytometry analysis with FlowJo software. Scatter signals and DAPI fluorescence were used to exclude cell debris and dead cells from the analysis. The DC purity was > 90%.


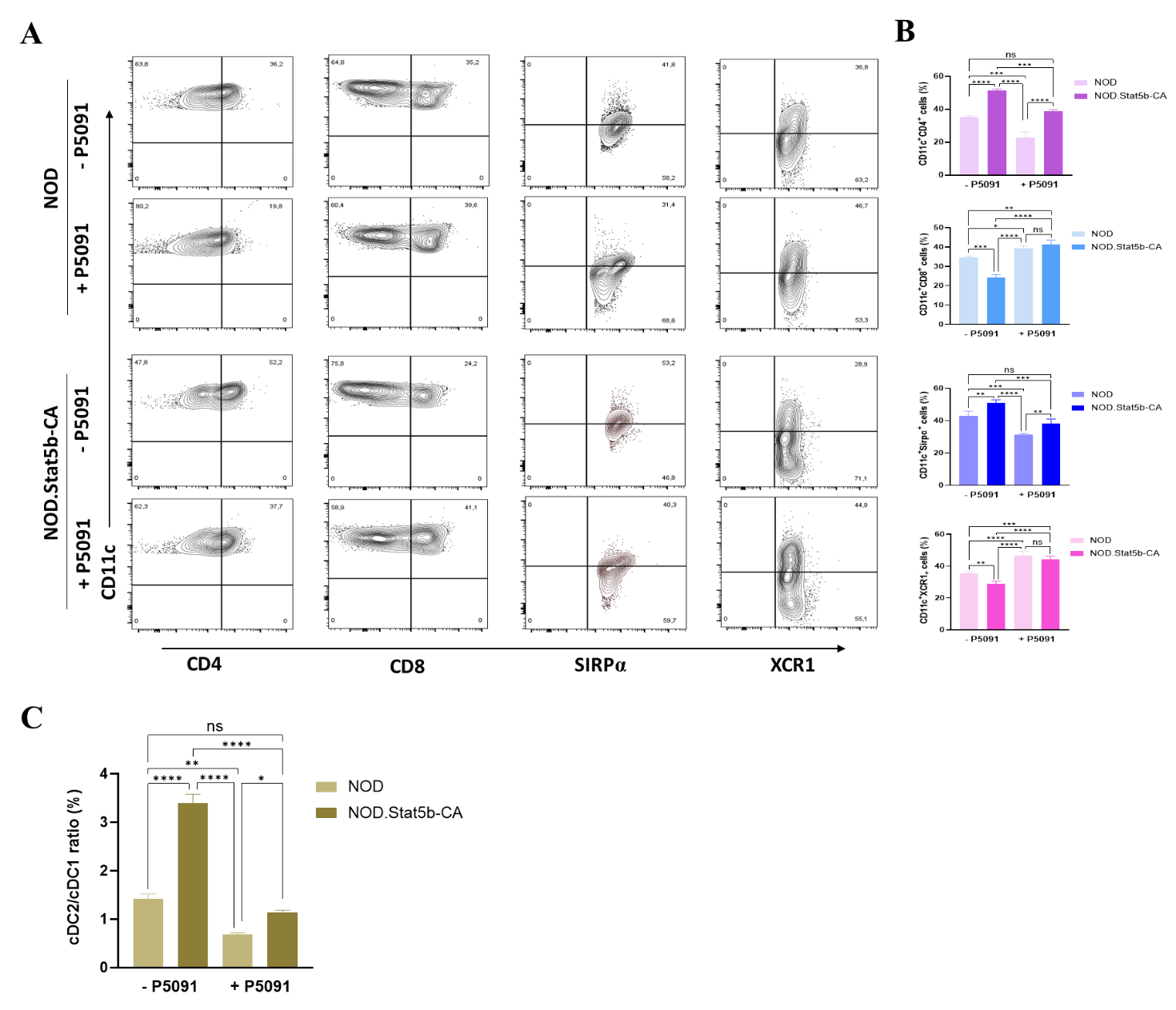


**Supplementary figure 2. Effect of USP7 blockade on cDC1 and cDC2 subsets expression and absolute number in NOD and NOD.Stat5b-CA mice.** Splenic DCs were purified from NOD and NOD.Stat5B mice and then treated with (+ P5091) or without (- P5091) USP7 inhibitor (5 µM/mL) for 24 hours. Thereafter, cells were washed, stained with respective mAbs, and analyzed by flow cytometry. (A) FACS profile of tolerogenic cDC2 (CD11c^+^CD4^+^Sirpα^+^) and immunogenic cDC1 (CD11c^+^CD8^+^XCR1^+^) cell subsets. (B) Representative bar graphs of CD11c^+^ DCs expressing CD4, CD8, Sirpα, and XCR1 molecules in NOD and NOD.Stat5b-CA mice. (C) The ratio of cDC2 and cDC1 in mice strains under study. Data are shown as the mean ± SEM of at least three independent experiments. The significance was calculated using One-way ANOVA with Tukey’s post-hoc test. n.s, not significant; *P ˂ 0.05; **P ˂ 0.01; ***P ˂ 0.001; ****P ˂ 0.0001.

**
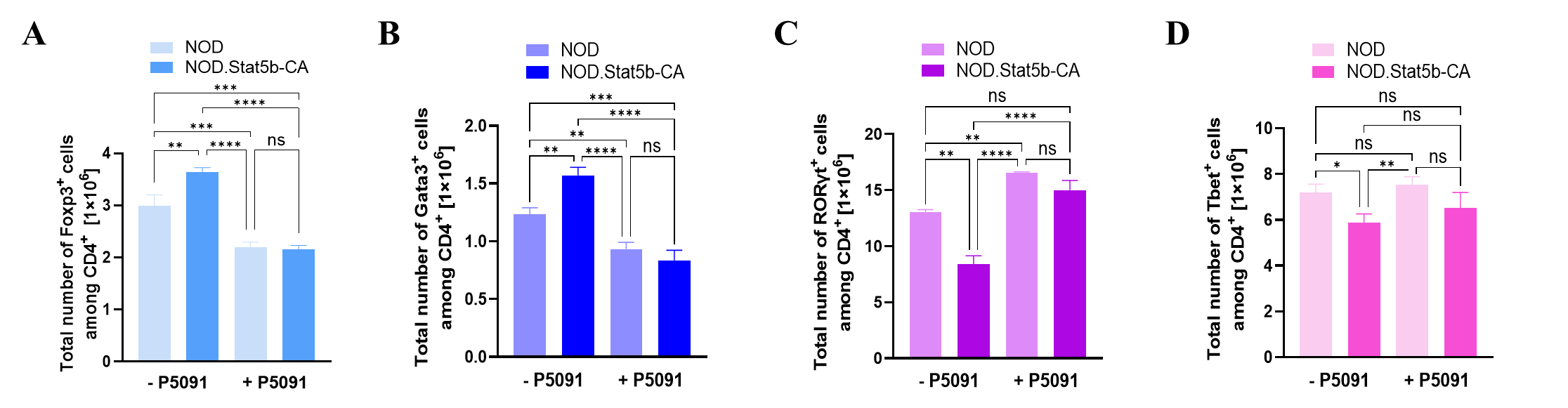
**

**Supplementary figure 3. USP7 blockade reduces the total numbers of Th2 and Treg cells while promoting Th17 and Th1 cells in vivo.** Purified splenic DCs from NOD and NOD.Stat5b-CA mice were pre-incubated with (+ PD5091) or without (- PD509) USP7 inhibitor (5 µM/mL) for 24 hours and then stimulated with LPS (1 µg/mL) for additional 24 hours. Cells were then washed, and 6×10^6^ cells were i.v injected into 8-10 weeks old NOD mice. After 7 days, spleen cells were harvested and analyzed for the total numbers of Tregs and Th1/Th2/Th17 cell subsets by FACS. The bar graph shows the absolute numbers of CD4^+^Foxp3^+^ (A), CD4^+^Gata3^+^ (B), CD4^+^RORγt^+^ (C), and CD4^+^Tbet^+^ (D) subsets. In all experiments, 4 mice per group were used, and the results are expressed as mean ± SEM. The significance was calculated using One-way ANOVA with Tukey’s post-test. n.s., not significant; *P ˂ 0.05; **P ˂ 0.01; ***P ˂ 0.001; ****P ˂ 0.0001.
